# Cholesterol‐Mediated Metabolic‐mechanotransductive Crosstalk Orchestrates Castration Resistance in Prostate Cancer

**DOI:** 10.1002/advs.75977

**Published:** 2026-06-04

**Authors:** Shaojie Liu, Chao Xu, Jun Jiang, Limin He, Yike Zhou, Zhengxuan Li, Yu Li, Keying Zhang, Fa Yang, Tong Lu, Hongtao Song, Hai Zhu, Zhihao Hu, Xiaolong Zhao, Kai Gan, Hongji Li, Bo Yang, Rui Zhang, Weihong Wen, Donghui Han, Weijun Qin

**Affiliations:** ^1^ Department of Urology Xijing Hospital Fourth Military Medical University Xi'an China; ^2^ Department of Health Service Base of Health Service Fourth Military Medical University Xi'an China; ^3^ Department of Urology Daping Hospital Army Medical University Chongqing China; ^4^ State Key Laboratory of Cancer Biology Department of Immunology Fourth Military Medical University Xi'an China; ^5^ School of Life Science and Technology Northwestern Polytechnical University Xi'an China

**Keywords:** cancer associated fibroblast, cholesterol, CRPC, matrix stiffness, mechanotransduction

## Abstract

Biophysical microenvironment fuels therapeutic resistance, yet the contribution of matrix stiffness to castration‐resistant prostate cancer (CRPC) remains poorly understood. In this study, we established a metabolic‐mechanotransductive crosstalk wherein cholesterol‐driven stromal reprogramming amplifies CRPC progression. Mechanistically, full androgen deprivation (FAD) induces cholesterol metabolic rewiring in prostate cancer (PCa) cells that orchestrates the CH25H‐dependent phenotype transformation of cancer‐associated fibroblast (CAF) into myofibroblastic CAF (myCAF). In turn, the resulting matrix stiffness induces unfolded protein response (UPR) and potentiates IRE1α kinase activity for *Xbp1* splicing, while concurrently activating the integrin αVβ3/FAK/STAT3 axis to transcriptionally replenish *Xbp1* substrate in PCa cells. This mechanosensitive adaptation thereby confers PCa with resistance to apoptosis induced by FAD. Consequently, pharmacological disruption of this metabolic‐mechanotransductive axis by targeting cholesterol metabolism or blockade of IRE1α‐XBP1s signaling significantly suppress tumor growth, representing a promising therapeutic strategy for CRPC progression.

## Graphical abstract

Full androgen deprivation (FAD) induces paracrine cholesterol in prostate cancer that drives the polarization of cancer‐associated fibroblasts (CAFs) and subsequent elevated matrix stiffness. Matrix stiffness in turn potentiates tumor cell survival under FAD pressure via dual mechanotransductive activation of the IRE1α‐XBP1s stress‐response axis.

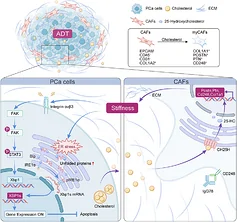

## 1 Introduction

Androgen receptor (AR) signaling is a critical oncogenic driver in prostate cancer (PCa), with AR signaling inhibitors (ARSIs) established as the first‐line systemic therapy for locally advanced and metastatic disease [1, 2]. Despite robust initial responses, intrinsic or acquired resistance inevitably emerges, driving progression to castration‐resistant prostate cancer (CRPC) and increased mortality [3]. PCa cells evade anti‐androgen therapy through reactivation of AR signaling or engagement of AR‐independent oncogenic pathways, ultimately leading to biochemical recurrence [4]. However, emerging evidence highlights the tumor microenvironment (TME) as a critical determinant in the progression to CRPC. Single‐cell RNA sequencing (scRNA‐Seq) analyses have unveiled a remarkably heterogeneous landscape in CRPC, with the TME displaying coordinated activation of transcriptome programs that orchestrate disease progression [5]. For instance, cancer‐associated fibroblasts (CAFs) promote anti‐androgen resistance via neuregulin 1 (NRG1)‐mediated HER3 activation, secreted phosphoprotein 1 (SPP1)‐mediated ERK signaling, and glucosamine‐driven androgen biosynthesis [6, 7, 8]. However, beyond transcription factors and metabolic components, the microstructural and functional implications of biophysical alterations remain poorly understood in CRPC progression.

PCa presents as a stroma‐rich solid malignancy. Clinically, digital rectal examination (DRE) remains a standard procedure for assessing prostate consistency. It provides preliminary differentiation between benign and malignant lesions based on matrix stiffness, which reflects alterations in the biomechanical properties of the TME [9]. The biophysical landscape is further characterized by features including solid stress, interstitial fluid pressure and flow, matrix stiffness, disordered tumor microstructure, and tissue viscoelasticity [10, 11]. Notably, matrix stiffness uniquely enables clinical palpation and facilitates diagnostic testing, synergizing with traditional examinations to enhance the accuracy and sensitivity in predicting tumor malignancy and prognosis [12]. Nonetheless, matrix stiffness exhibits marked heterogeneity in progression, prognosis, and particularly therapeutic resistance across tumor types. For instance, elevated matrix stiffness drives platinum‐drug resistance in ovarian cancer, bevacizumab resistance in colon cancer liver metastases, and promotes immunotherapy tolerance in multiple tumors, while paradoxically enhancing paclitaxel efficacy in breast cancer [13, 14, 15, 16]. Prior evidence demonstrates elevated matrix stiffness in PCa compared to benign prostatic hyperplasia, correlating with poor prognosis [17]. Yet, its functional role in CRPC remains to be elucidated. Mechanistically, matrix stiffness is fueled by the activation and proliferation of CAFs, coupled with pathological ECM remodeling [10, 18]. Accumulating evidence highlights CAFs as pivotal stromal effectors in CRPC progression. While the paracrine pro‐tumor function of CAFs in CRPC are established, their mechanoregulatory roles in shaping the biomechanical niche of CRPC remain unexplored.

Here, we identify a metabolic‐mechanotransductive crosstalk that orchestrates therapeutic resistance in PCa. Androgen deprivation therapy (ADT) triggers metabolic reprogramming in PCa cells, promoting enhanced cholesterol biosynthesis and secretion. Upon uptake by CAFs, cholesterol is hydroxylated to generate 25‐hydroxycholesterol (25‐HC). This oxysterol acts as a potent inducer of myofibroblastic CAF (myCAF) differentiation resulting in extensive ECM deposition and matrix stiffening. In turn, matrix stiffness induces endoplasmic reticulum stress (ERS) and activates integrin αVβ3/FAK/STAT3 signaling in PCa cells. This mechanosensitive adaptation promotes IRE1α‐dependent *Xbp1* splicing, thereby conferring resistance to apoptosis induced by ADT. Collectively, our findings reveal that cholesterol‐driven stromal reprogramming and mechanotransduction govern CRPC progression, highlighting the critical interplay between metabolism and biomechanics in therapeutic resistance.

## 2 Results

### 2.1 Matrix Stiffness Significantly Increases in CRPC

**FIGURE 1:**
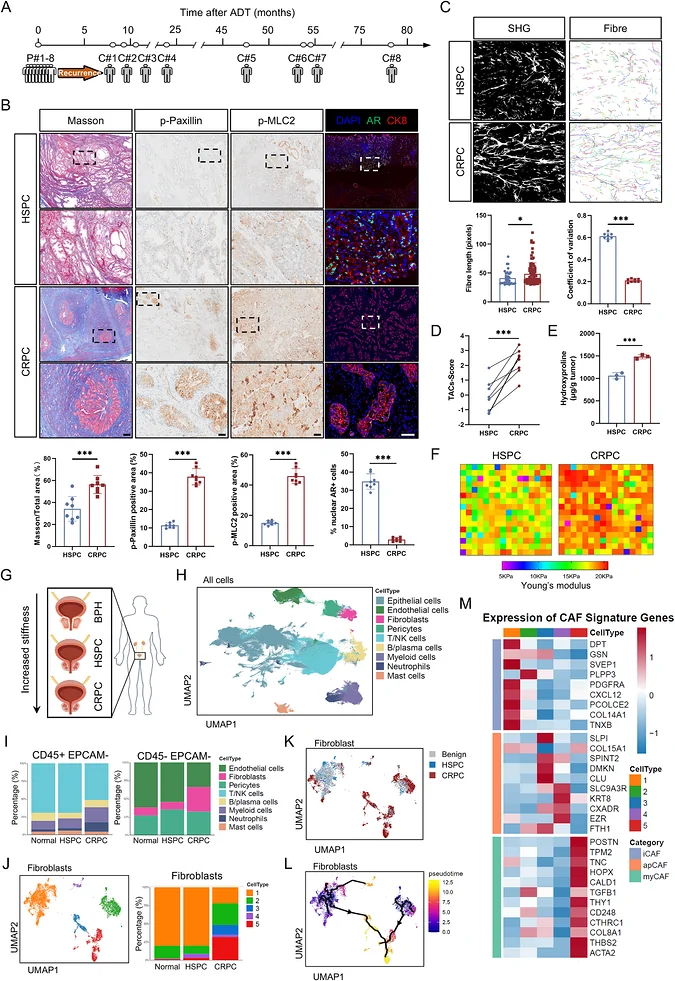
Matrix stiffness significantly increases in CRPC. (A) Schematic of clinical course from HSPC to CRPC in paired specimens from the same patients (n = 8). Local recurrent PCa tissues that occurred after the initial surgery were collected from a cohort of paired patients who received adjuvant therapy with antiandrogens. (B) Representative images and quantification of Masson staining for collagen, immunohistochemical (IHC) staining for p‐Paxillin and p‐MLC2, and immunofluorescence (IF) staining for AR (n = 8). Scale bar, 100 µm. (C) Representative second harmonic generation (SHG) imaging via two‐photon microscopy revealing deep collagen architecture, with quantitative analysis of fiber density and alignment (n = 8). (D) Tumor‐Associated Collagen Signature (TACS) scoring and statistical comparison between groups (n = 8). (E) Hydroxyproline quantification assay demonstrating collagen content in fresh tumor tissues (n = 3). (F) Representative atomic force microscopy (AFM)‐based mapping of Young's modulus distribution across tumor regions. (G) Schematic of stiffness increase in prostate. (H) UMAP visualization of cellular clustering from single‐cell sequencing data. (I) Compositional distribution of CD45^−^EPCAM^−^ and CD45^+^EPCAM^−^ cellular subsets across specimens. (J) UMAP visualization of re‐clustered fibroblasts populations with the proportional representation across clinical specimens. (K) UMAP visualization of disease stage‐specific spatial distribution patterns of fibroblast subpopulations. (L) Pseudotime trajectory analysis of diverse CAF subpopulations using Monocle 3. (M) Heatmap depicting expression profiles of signature genes for inflammatory CAFs (iCAFs), antigen‐presenting CAFs (apCAFs), and myofibroblast‐like CAFs (myCAFs) across fibroblast subsets. *p < 0.05, ***p < 0.001.

To interrogate the alterations of physical properties in CRPC, we analyzed specimens from 8 paired PCa patients and established spontaneous PCa in PTEN/Trp53 double knockout (DKO) mice (Figure 1A and S1A). CRPC induction via ARSIs or castration followed by enzalutamide (ENZ) gavage resulted in reduced AR nuclear localization, as visualized by immunofluorescence (IF; Figure 1B and S1B). Masson's trichrome staining revealed heightened collagen deposition, while immunohistochemical analysis demonstrated elevated phosphorylation of Paxillin and myosin light chain 2 (MLC2), canonical hallmarks of enhanced mechanotransduction in CRPC (Figure 1B and S1B). Second harmonic generation (SHG) imaging via two‐photon microscopy further confirmed increased collagen content, fiber length and Tumor‐Associated Collagen Signature (TACS) scores with reduced variation coefficients in CRPC (Figure 1C,D and S1C–E), corroborated by hydroxyproline quantification (Figure 1E and S1F). Atomic force microscopy (AFM) directly validated stiffness through augmented surface roughness and Young's modulus in CRPC (Figure 1F; S1G,H and S2A). Collectively, these data establish increased matrix stiffness as a hallmark of CRPC, reflecting pathological remodeling of the glandular ECM architecture (Figure 1G).

We next performed integrated analysis of the scRNA‐seq data from 9 PCa datasets to dissect TME dynamics underlying this biomechanical reprogramming (Table S1) [5, 19, 20, 21, 22, 23, 24, 25, 26]. After stratifying the TME into 9 cell subgroups based on marker expression and quantifying abundance shifts (Figure 1H and S2B), we observed marked enrichment of CAFs within the CD45^−^EPCAM^−^ stromal compartment in CRPC, whereas other stromal and immune populations remained unaltered compared to hormone‐sensitive prostate cancer (HSPC; Figure 1I). Fibroblasts re‐clustering identified 5 distinct subsets, with Cluster 5 uniquely present in CRPC (Figure 1J,K and S2C‐D). Pseudotime trajectory analysis predicted lineage progression wherein cluster 5 arises from the other CAF subsets (Figure 1L). Functional characterization and cluster‐specific differentially expressed genes (DEGs) profiles identified Cluster 5 as a myCAF population (Figure 1 M and S2E). Notably, StromalScore of the DEG profiles derived from Gene Expression Omnibus (GEO) and The Cancer Genome Atlas (TCGA) datasets was elevated in CRPC and correlated with shorter progression‐free survival following ARSIs treatment (Figure S2F,G). Together, these findings reveal that myCAFs critically remodel the ECM through increased collagen deposition and matrix stiffness to biomechanically support CRPC progression.

### 2.2 CD248^+^ CAFs Drive Matrix Stiffening and Castration Resistance in Prostate Cancer

**FIGURE 2:**
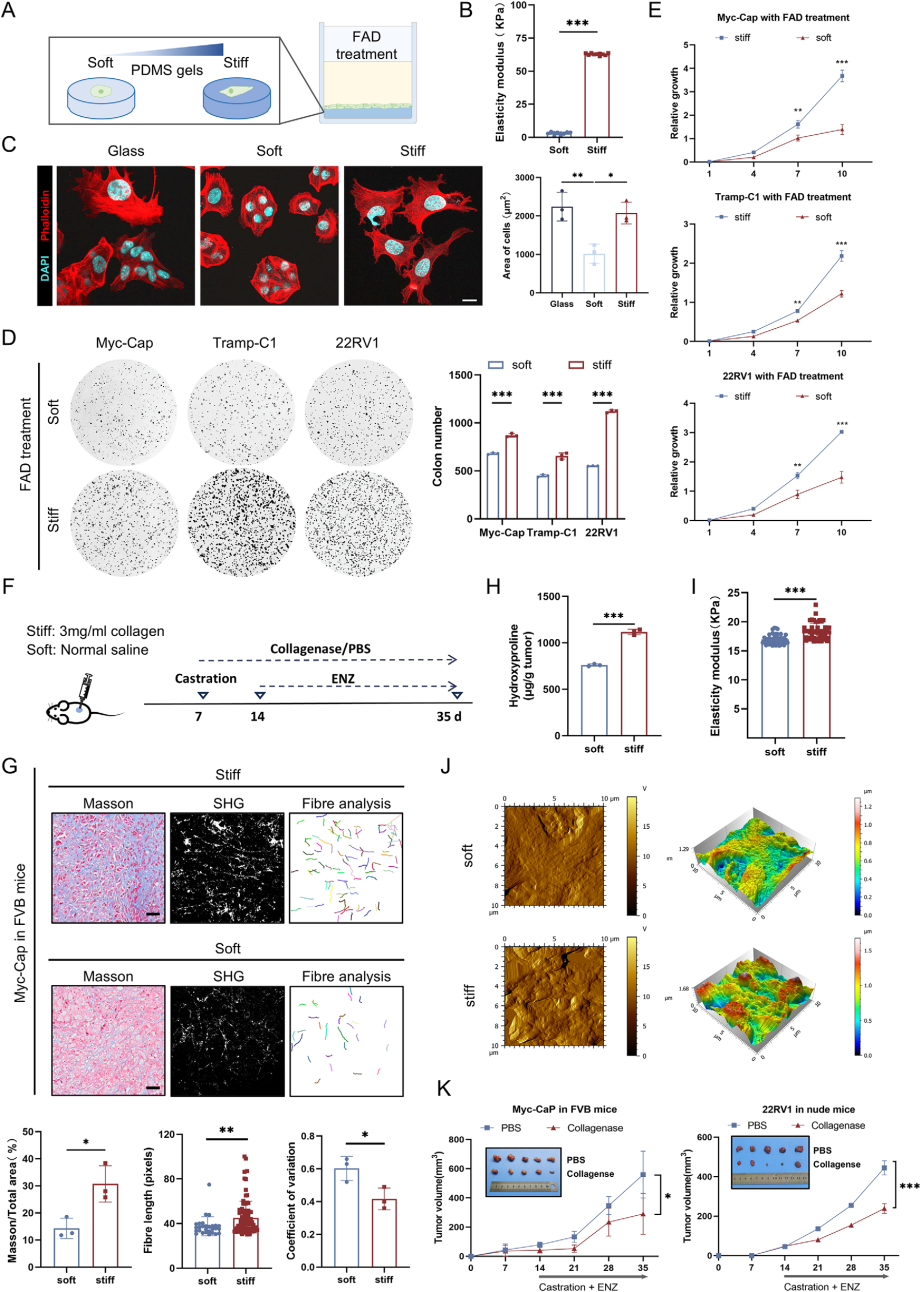
Matrix stiffness promotes the progression of CRPC. (A) Schematic illustration of the construction of PDMS‐based cell culture substrates. (B) Statistical analysis of Young's modulus of cell culture substrates measured by AFM (n = 10). Scale bar, 20 µm. (C) Representative images and statistical analysis of cytoskeleton immunofluorescence staining in soft and stiff substrates (n = 3). (D) Statistical analysis of the number and size of PCa cell colonies in soft and stiff substrates (n = 3). (E) Proliferation curves of PCa cells in soft and stiff substrates over time (n = 3). (F) Schematic illustration of the construction of tumor‐bearing mouse models with soft and stiff matrix. (G) Representative images and statistical analysis of Masson and collagen network imaging in PCa tissues (n = 3, bar = 100 µm). (H) Detection of hydroxyproline content in PCa tissues (n = 3). (I) Statistical analysis of the elastic modulus of PCa tissues measured by AFM (n = 5, 10 points per sample). (J) Representative images of PCa tissue morphology measured by AFM (n = 3). (K) Tumor images and growth curves of PCa volume inoculated subcutaneously in mice (n = 5). *p < 0.05, **p < 0.01, ***p < 0.001.

To mechanistically dissect the contribution of matrix stiffness to CRPC progression, we engineered polydimethylsiloxane (PDMS)‐based substrates with tunable rigidity. Modulation of prepolymer‐crosslinker ratios, validated by AFM, established conditions mimicking soft (10 kPa) and stiff (60 kPa) matrices (Figure 2A,B). Confocal microscopy revealed stiffness‐dependent morphological polarization: PCa cells extended pseudopodia and displayed cytoskeletal spreading on stiff substrates but formed spheroidal clusters on soft substrates (Figure 2C). Colony formation assays under FAD conditions demonstrated significantly increased proliferation on stiff substrates (Figure 2D,E). For in vivo interrogation, we generated CRPC xenografts with contrasting biomechanical properties: collagen co‐injection (stiff) versus collagenase‐mediated peritumoral softening (soft) [27] (Figure 2F). Stiff tumors exhibited desmoplastic remodeling hallmarks including elevated collagen deposition (Masson's trichrome/SHG; Figure 2G and S3A), increased hydroxyproline content (Figure 2H and S3B), heightened surface roughness and elevated Young's modulus (AFM; Figure 2I‐J and S3C), collectively accelerating tumor growth (Figure 2K). These data indicate that matrix stiffness autonomously regulates neoplastic behavior within the TME, independent of CAF‐paracrine signaling. To deconvolute CAF‐independent effects, we established a transwell co‐culture system utilizing fluorescence‐activated cell sorting (FACS)‐isolated EPCAM^−^CD31^−^CD45^−^ CAFs and tumor cells on PDMS substrates (Figure S3D). Colony formation assays confirmed that while CAF co‐culture enhanced proliferation, stiffness‐dependent amplification persisted: stiff substrates consistently augmented colony formation compared to soft substrates under identical CAF exposure (Figure S3E,F).

**FIGURE 3:**
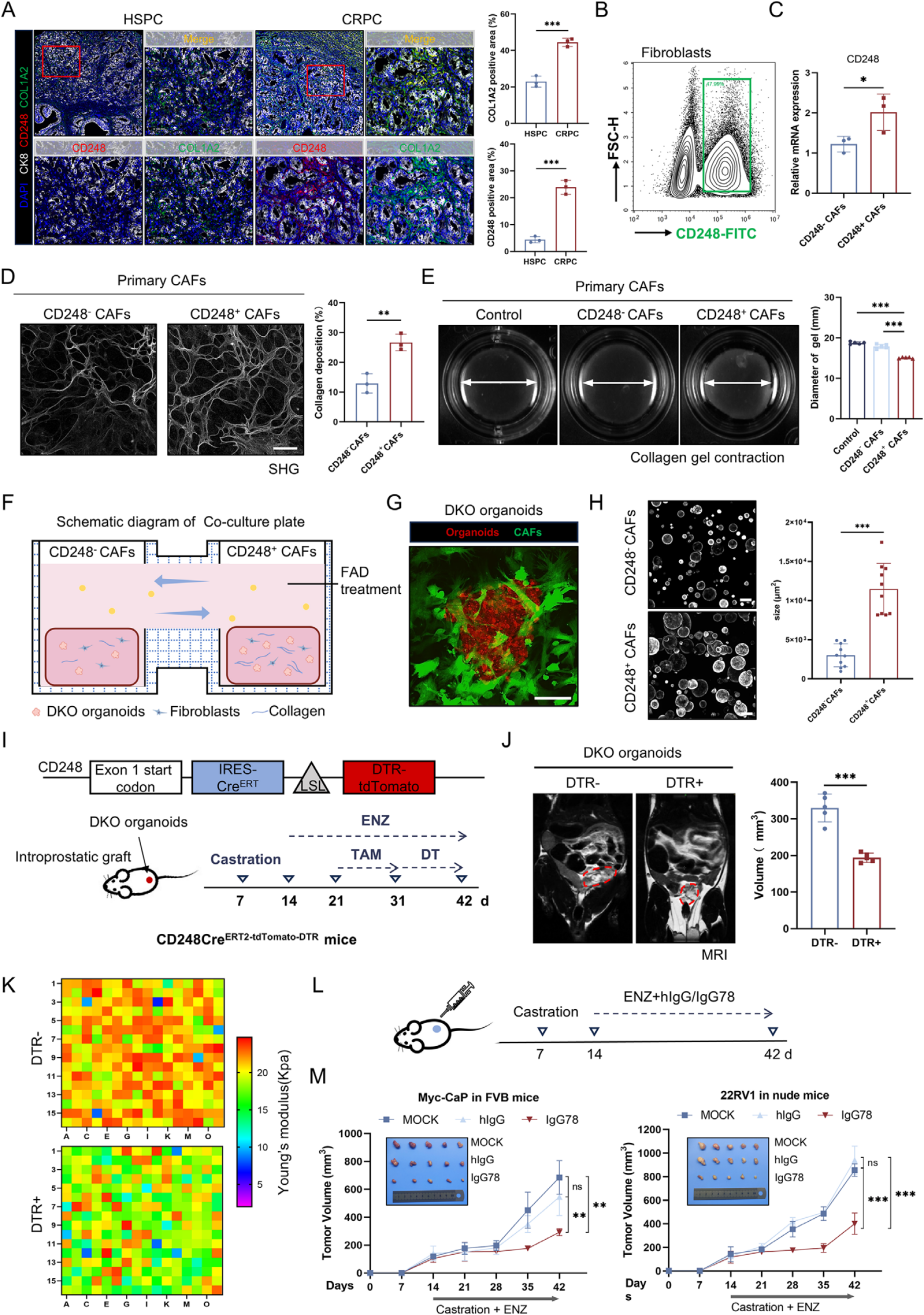
CD248^+^ CAFs drive matrix stiffening and castration resistance in prostate cancer. (A) Representative multicolor IF staining images of the expression and distribution of CD248 in PCa, with quantification (n = 3). Scale bar, 100 µm. (B) Flow cytometry gating strategy for isolation of CD248^−^ CAFs and CD248^+^ CAFs. (C) RT‐qPCR validation of CD248 mRNA levels in CAFs (n = 3). (D) Representative SHG images of ECM content and structure organization secreted by CAFs in cell slides, with quantification (n = 3). Scale bar, 20 µm. (E) Representative images of collagen gel contraction assay demonstrating CAF‐mediated matrix remodeling, with quantitative analysis (n = 5). (F) Schematic illustration of CAF‐organoid co‐culture system. (G) Representative spinning disk confocal microscopy images of CAF‐organoid interactions. Organoids were labelled by mCherry and CAFs by EGFP. Scale bar, 20 µm. (H) Representative spinning disk confocal microscopy images of organoids co‐cultured with CAFs, with quantification of organoid growth/ morphology (n = 10). Scale bar, 20 µm. (I) Schematic of CD248^CreERT‐tdTomato‐DTR^ mouse generation and therapeutic workflow. (J) Representative MRI images of in situ prostate cancer xenografts in CD248^CreERT‐tdTomato‐DTR^ mice, with quantification of tumor volume (n = 5). (K) Representative AFM images showing tissue topography of in situ tumor xenografts in CD248^CreERT‐tdTomato‐DTR^ mice (n = 5). (L) Schematic of mouse xenograft model establishment and combined treatment regimen. (M) Representative images of subcutaneous PCa xenografts and corresponding tumor volume growth curves (n = 5). **p < 0.01, ***p < 0.001.

Given the scRNA‐seq identification of myCAFs as primary ECM‐remodeling effectors in CRPC, we sought selective markers to disrupt their biomechanical function. Transcriptional profiling identified CD248, an established marker of ECM deposition in fibrotic disease and malignancies [28], as a distinguishing myCAF marker (Figure 1 M, S4A). Multiplex immunofluorescence validated CD248^+^/COL1A2^+^ co‐localization in CRPC specimens (Figure 3A). FACS isolated murine CD248^+^ CAFs, with expression confirmed by RT‐qPCR and immunoblotting (Figure 3B,C and S4B), demonstrated superior matrix remodeling compared to CD248^−^ counterparts. Decellularization assays visualized by two‐photon microscopy revealed that CD248^+^ CAF generate denser, aligned fibrillar networks following 7‐day culture (Figure 3D), while collagen gel contraction assays confirmed enhanced contractility (Figure 3E). In horizontal co‐culture system to eliminate paracrine effects, CD248^+^ CAFs structurally integrated with *Pten/Trp53* DKO mice‐derived organoids (Figure 3F,G), driving proliferation (Figure 3H and S4C) via ECM‐mediated mechanoregulation.

To establish CD248^+^ CAFs‐specific ablation, we engineered *CD248^CreERT2‐tdTomato‐DTR^* mice bearing orthotopic CRPC organoid xenografts. Tamoxifen‐induced Cre recombinase enabled tdTomato labeling and diphtheria toxin receptor (DTR) expression, permitting targered depletion (Figure 3I and S4D). DT‐treated mice exhibited suppressed tumor growth (Figure 3J and S4E), reduced collagen deposition, disrupted fibrillar networks (Figure S4F–I) and diminished matrix stiffness evidenced by decreased surface roughness and Young's modulus (Figure 3K and S4J,K), establishing CD248^+^ CAFs as master regulators of CRPC biomechanics. Consequently, CD248‐targeting monoclonal antibody IgG78 synergized with ADT to suppress CRPC progression, identifying CD248^+^ CAFs as actionable mechanotherapeutic targets (Figure 3L,M).

### 2.3 Matrix Stiffness Sustains ADT Resistance in PCa Through IRE1α‐XBP1s Activation

**FIGURE 4:**
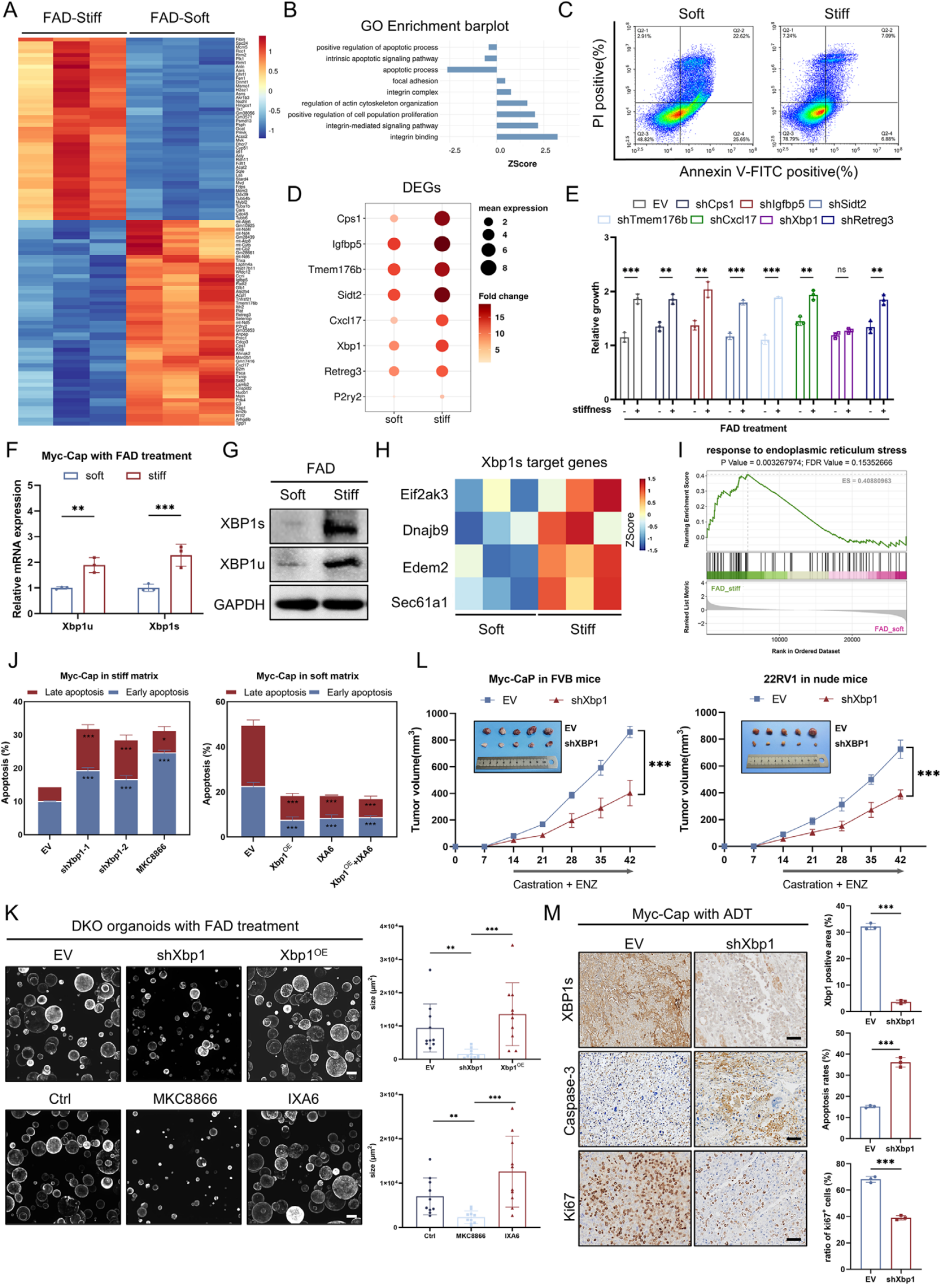
Matrix stiffness sustains ADT resistance in PCa through IRE1α‐XBP1s activation. (A) Heatmap of the top 100 DEGs in Myc‐Cap cells on the soft and stiff substrates treated with FAD treatment (n = 3). (B) GO enrichment analysis of the DEGs. (C) Representative images of flow cytometry detecting apoptosis of Myc‐Cap cells on the soft and stiff substrates with FAD treatment (n = 3). (D) Bubble plot of growth‐related DEGs among the top 50 DEGs in Myc‐Cap cells on the soft and stiff substrates under FAD treatment. (E) Statistical analysis of the growth of Myc‐Cap cells with shRNA knockdown of the target genes (n = 3). (F) RT‐qPCR analysis of Xbp1u and Xbp1s expression in Myc‐Cap cells on the soft and stiff substrates under FAD treatment (n = 3). (G) Immunoblotting analysis of Xbp1u and Xbp1s expression in Myc‐Cap cells on the soft and stiff substrates under FAD treatment. (H) Heatmap comparing the scaled normalized expression of XBP1s downstream genes in Myc‐Cap cells on the soft and stiff substrates (n = 3). (I) GSEA analysis of DEGs in “response to endoplasmic reticulum stress”. (J) Statistical analysis of apoptosis of Myc‐Cap cells detected by flow cytometry (n = 3). (K) Representative images of organoids derived from spontaneous prostate cancer mice under a spinning disk confocal microscope and statistical analysis of their sizes (n = 10). Scale bar, 20 µm. (L) Tumor images and the growth curves of subcutaneous xenografts of EV and Xbp1 KD PCa cells in mice (n = 5). (M) Representative images and statistical analysis of IHC staining of tumor tissues from subcutaneous xenografts of EV and Xbp1 KD Myc‐Cap cells in mice. Scale bar, 100 µm. **p < 0.01, ***p < 0.001.

While matrix stiffness drives CRPC progression, its mechanistic basis remains undefined. To interrogate potential engagement of AR signaling or neuroendocrine prostate cancer (NEPC) differentiation, we quantified AR targets (*Fkbp5, Trpm8, Nkx3.1, Slc45a3*) and NEPC markers (*Ezh2, Eno2, Chga, Syp, Ascl1*) in FAD‐treated Myc‐Cap cells. RT‐qPCR revealed stiffness‐independent alterations (Figure S5A,B), excluding AR pathway dependence and transdifferentiation as mechano‐resistant mediators. To dissect stiffness‐driven castration resistance, Myc‐Cap cells on rigidity‐tuned PDMS underwent FAD treatment followed by RNA‐seq. Principal component analysis (PCA) revealed distinct FAD‐response clustering (Figure S5C), identifying 2,885 stiffness‐dependent DEGs. Hierarchical clustering of the top 100 DEGs showed substrate‐specific reprogramming with 53 transcripts upregulated on stiff substrates (Figure 4A). GO enrichment demonstrated negative regulation of apoptosis (“positive regulation of apoptotic process”, “intrinsic apoptotic signaling pathway”, “apoptotic process”) and positive enrichment of cytoskeletal/integrin signaling (“focal adhesion”, “integrin complex”, “regulation of actin cytoskeleton organization”) (Figure 4B). Flow cytometry confirmed stiffness‐dependent apoptosis suppression that Annexin V/PI staining showed significant apoptosis on soft substrates versus minimal cell death on stiff substrates under FAD treatment (Figure 4C).

Among top 50 upregulated DEGs, we identified XBP1 as a critical mechanoresponsive driver. shRNA knockdown confirmed that *Xbp1* silencing suppressed proliferation exclusively on stiff substrates, whereas other maintained growth advantages (Figure 4D,E and S5D). Stiffness upregulated both unspliced (*Xbp1u*) and spliced (*Xbp1s*) isoforms (Figure 4F,G), activating XBP1s‐target genes (*Eif2ak3, Dnajb3, Edem2, Sec61a1*; Figure 4H) and ERS response enrichment (Figure 4I). Toyocamycin‐mediated inhibition of *Xbp1* splicing abolished stiffness‐dependent survival (Figure S5E), confirming its necessity. GSEA further established matrix stiffness as a positive regulator of ERS under FAD treatment: FAD suppressed ERS on soft substrates while stiffness maintained its activity independently (Figure S5F). RT‐qPCR/immunoblotting confirmed stiffness‐dependent ERS modulation that matrix stiffness upregulated IRE1α phosphorylation and *Xbp1s*/*Xbp1u*, with FAD treatment downregulating *Xbp1s* on soft substrates but sustaining signaling on stiff substrates (Figure S5G,H). We also examined the expression of PERK/eIF2α and ATF6 as additional marker panel of ER stress, while the negative results indicated that IRE1α is the specific sensor activated by stiffness (Figure S5I). Collectively, matrix stiffness reactivates FAD‐suppressed ERS through IRE1α‐XBP1s pathway restoration, defining a mechanotransductive bypass of hormonal therapy.

To define IRE1α‐XBP1s signaling hierarchy, we engineered Myc‐Cap cells with validation via RT‐qPCR/immunoblotting (Figure S5J,K). Apoptosis analysis revealed stiffness‐dependent resistance: *Xbp1* knockdown or RNase inhibitor MKC8866 sensitized cells to FAD‐induced apoptosis on stiff substrates, whereas XBP1 overexpression or agonist IXA6 attenuated apoptosis on soft substrates (Figure 4J and S5L). In Pten/Trp53 DKO organoids, XBP1 suppression (knockdown/MKC8866) inhibited while activation (overexpression/IXA6) enhanced FAD‐resistant proliferation (Figure 4M). In vivo, XBP1 inhibition synergized with ADT to suppress tumor progression (Figure 4L and S5M), with reduced tumors showing elevated cleaved caspase‐3 and reduced Ki67 (Figure 4M). Thus, matrix stiffness confers apoptosis resistance via IRE1α‐XBP1s activation, establishing biomechanical ERS regulation as a therapeutic vulnerability in CRPC.

### 2.4 Matrix Stiffness Promotes XBP1 Expression via Integrin αVβ3/FAK/STAT3 Signaling Pathway

**FIGURE 5:**
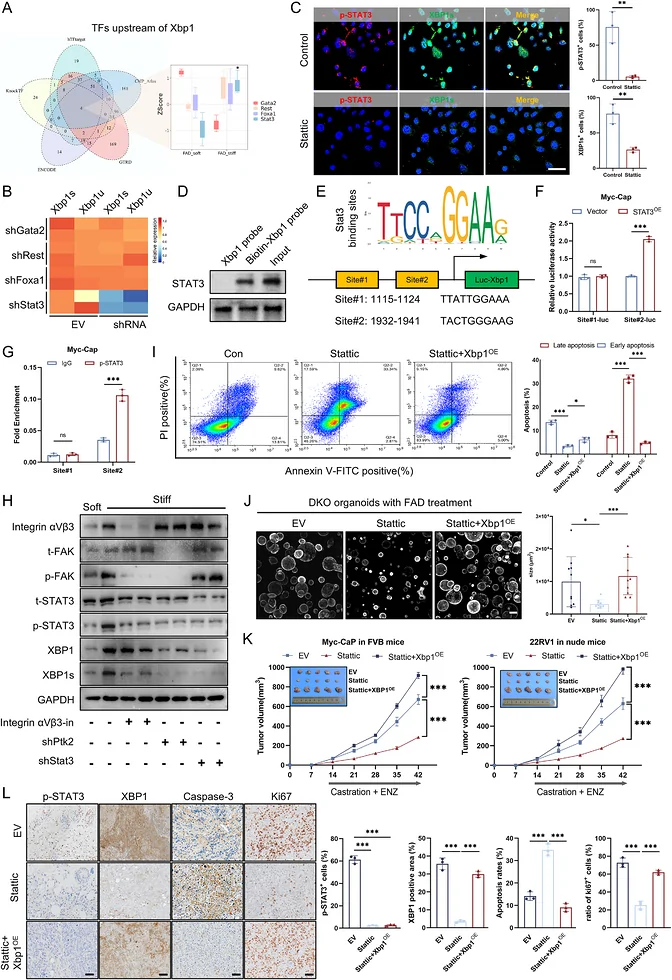
Matrix stiffness promotes XBP1 expression via Integrin αVβ3/FAK/STAT3 signaling pathway. (A) Screening of upstream transcription factors of Xbp1 among the ChIP‐seq databases and their expression in Myc‐Cap cells cultured on soft and stiff substrates. (B) RT‐qPCR analysis of Xbp1u and Xbp1s expression in Myc‐Cap cells with knockdown of the target genes under FAD treatment (n = 3). (C) Representative images of IF staining to detect the nuclear localization of p‐STAT3 and XBP1s in Myc‐Cap cells (n = 3). Scale bar, 20 µm. (D) Immunoblotting analysis of the binding of STAT3 to the Xbp1 probe. (E) Motif sequence map of the binding sites between STAT3 and the Xbp1 promoter in mouse‐derived cells predicted by the JASPAR website. (F) Statistical analysis of luciferase activity at the binding sites between STAT3 and the Xbp1 promoter detected by the dual‐luciferase reporter gene assay (n = 3). (G) Statistical analysis of the amplification of the Xbp1 promoter sequence bound to STAT3 detected by ChIP‐qPCR (n = 3). (H) Immunoblotting analysis of the regulation of XBP1 expression by the Integrin αVβ3/FAK/STAT3 signaling pathway in Myc‐Cap cells. (I) Representative images of flow cytometry detection of apoptosis in Myc‐Cap cell with FAD treatment (n = 3). (J) Representative images of organoids derived from spontaneous prostate cancer mice under a spinning‐disk confocal microscope and statistical analysis of their sizes (n = 10). Scale bar, 20 µm. (K) Images of subcutaneous tumors and the growth curves of volume in mice bearing EV or XBP1‐overexpressing PCa cells with or without Stattic treatment (n = 5). *p < 0.05, ***p < 0.001.

Although AR historically activates *Xbp1* transcription [29], we observed that ENZ suppressed XBP1 expression that was counteracted by matrix stiffness under FAD treatment, revealing an AR‐independent compensatory axis. Integration of five ChIP‐seq repositories (hTFtarget/ChIP_Atlas/GTRD/ENCODE/KnockTF) nominated GATA2, REST, FOXA1, and STAT3 as putative *Xbp1* regulators (Figure 5A; Table S2). Transcriptomics revealed stiffness‐dependent divergence that *Stat3* upregulated on stiff substrates with *Gata2* downregulated (Figure 5A). Functional validation via shRNA knockdown confirmed STAT3‐specific control of *Xbp1* isoforms, as *Stat3* depletion reduced both unspliced and spliced isoforms (Figure 5B; S6A). Mechanistically, stiffness‐induced STAT3 phosphorylation (p‐STAT3) correlated with XBP1s/u upregulation (Figure S6B); Stattic inhibition blocked p‐STAT3 nuclear translocation and XBP1s expression (Figure 5C). Genomic analysis confirmed STAT3 binding at the Xbp1 promoter (Figure S6C), establishing phosphorylation‐dependent nuclear accumulation as the transcriptional orchestrator of IRE1α‐XBP1s signaling.

**FIGURE 6:**
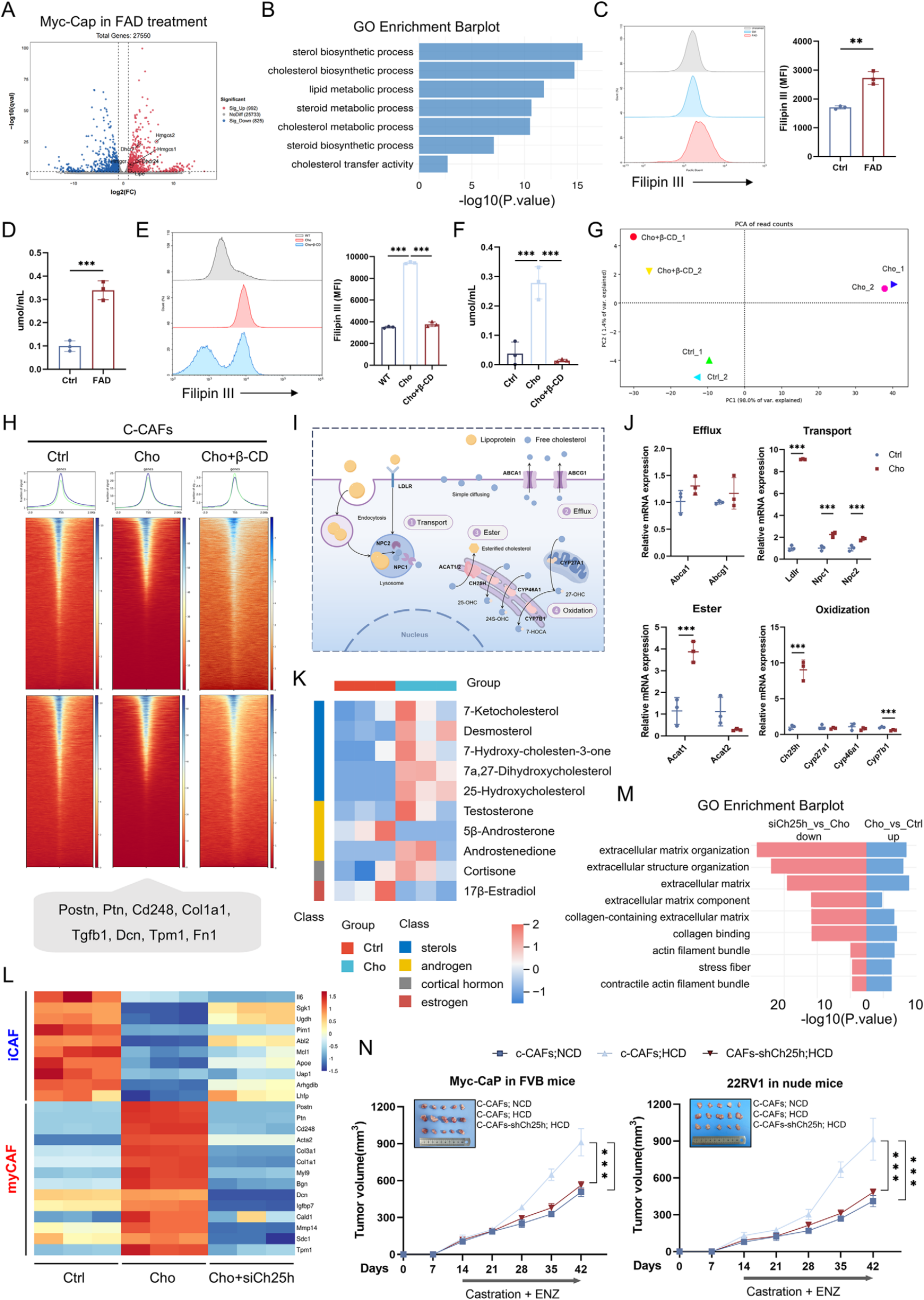
CH25H‐mediated cholesterol metabolic rewiring in CRPC drives CAFs polarization. (A) Volcano plot of DEGs in FAD‐treated Myc‐Cap cells on soft substrates. (B) GO enrichment analysis of the DEGs identified in (A). (C) Flow cytometry analysis of Filipin III MFI in Myc‐Cap cells with or without FAD treatment (n = 3). (D) Quantification of total cholesterol content in Myc‐Cap cells with or without FAD treatment (n = 3). (E) Flow cytometry analysis of Filipin III MFI in CAFs (n = 3). (F) Detection of total cholesterol content in CAFs (n = 3). (G) Principal component analysis (PCA) of the ATAC‐seq data across the CAF subsets. (H) Heatmap of ATAC‐seq profiles of indicated CAFs. (I) Schematic illstration of intracellular cholesterol trafficking. (J) RT‐qPCR anaysis of key cholesterol metabolism enzymes expression in CAFs with or without cholesterol treatment (n = 3). (K) Heatmap of targeted metabolomics sequencing of steroid hormones in CAFs. (L) Heatmap comparing the scaled normalized expression of iCAF and myCAF marker genes among indicated CAFs. (M) Indicated GO terms analysis of the DEGs across the CAF subsets. (N) Growth curves of PCa with ADT after subcutaneous co‐injection with CAFs or Ch25h KD CAFs under normal cholesterol diet (NCD) or HCD conditions (n = 5). **p < 0.01, ***p < 0.001.

To establish direct STAT3‐XBP1 regulation, we designed an Xbp1 promoter DNA probe. DNA pull‐down assays coupled with proteomics identified 1,459 bound proteins, including STAT3 verified by mass spectrometry and immunoblotting (Figure 5D; S6D). JASPAR‐based binding site prediction (Figures 6E; S6E,F) guided lentiviral STAT3 overexpression in Myc‐Cap/HEK293T cells (Figure S6G); dual‐luciferase and ChIP‐qPCR further precisely mapped functional STAT3 occupancy sites (Figure 6F,G; S6H,I). Given stiffness‐induced enrichment of integrin/focal adhesion pathways (Figure 4B), we interrogated integrin αVβ3/FAK/STAT3 signaling in XBP1 regulation. Pharmacological integrin αVβ3 inhibition or Ptk2/Stat3 knockdown suppressed p‐FAK with subsequent reduced p‐STAT3 and downregulated XBP1u/s isoforms (Figure 5H), validating this mechanotransduction axis.

Functionally, STAT3 governs IRE1α/XBP1s‐mediated apoptosis resistance in CRPC. Flow cytometry confirmed Stattic‐induced apoptosis in FAD‐treated Myc‐Cap cells, which was rescued by XBP1 overexpression (Figure 5I). Organoid/xenograft models demonstrated STAT3 inhibition suppressed tumor proliferation, reversible upon XBP1 reconstitution (Figure 5J,K; S6J). Mechanistically, matrix stiffness triggers Integrin αVβ3/FAK/STAT3 signaling, inducing STAT3 phosphorylation and nuclear translocation to bind the *Xbp1* promoter, thereby activating transcription and providing substrates for IRE1α‐mediated ERS response, ultimately driving castration resistance in prostate cancer.

### 2.5 Cholesterol Metabolic Rewiring in CRPC Drives CH25H‐Mediated CAFs Polarization

Tumor cells critically drive phenotypic transformation of CAFs. To investigate the underlying mechanisms, we performed RNA sequencing (RNA‐seq) on PCa cells subjected to FAD treatment on soft substrates. Analysis identified 4,853 upregulated and 4,487 downregulated DEGs (Figure 6A). Gene Ontology (GO) enrichment analysis revealed significant enrichment in cholesterol biosynthesis and metabolic process (Figure 6B). Key genes in the cholesterol synthesis and hydrolysis pathway (*Lipe*, *Hmgcr*, *Hmgcs1*, *Hmgcs2*, *Dhcr7*, *Dhcr24)* were markedly upregulated, consistent with Gene Set Enrichment Analysis (GSEA) validation of “cholesterol biosynthetic process” enrichment (Figure S7A,B). Flow cytometry and enzymatic assays demonstrated increased Filipin III mean fluorescence intensity (MFI) and total cholesterol levels in FAD‐treated PCa cells, confirming enhanced cholesterol accumulation (Figure 6C,D).

Conditioned medium (CM) from castration‐resistant PCa cells induced significant upregulation of myCAF markers (*Acta2*, *Col1a1*, *Cd248*, *Postn*) in CAFs by RT‐qPCR. Immunofluorescence further confirmed increased Filipin III MFI in these CAFs, which was abrogated by β‐cyclodextrin treatment (β‐CD; Figure S7C,D). Exogenous cholesterol treatment induced concentration‐dependent myCAF differentiation (Figure S7E,F). Flow cytometry and enzymatic assays confirmed active cholesterol uptake by CAFs, evidenced by increased Filipin III intensity and total cholesterol levels (Figure 6E,F). Assay for Transposase‐Accessible Chromatin sequencing (ATAC‐seq) further revealed increased chromatin accessibility at 63,438 sites in cholesterol‐treated CAFs relative to controls, while β‐CD treatment restored baseline accessibility (Figure 6G,H), indicating cholesterol promotes CAF polarization. Functional assays further revealed that cholesterol‐exposed CAFs exhibit enhanced ECM secretion and contractility, hallmarks of myCAFs (Figure S7G,H).

Cholesterol metabolism encompasses excretion, esterification, and oxidation, with oxidized metabolites modulating transcription [30] (Figure 6I). RT‐qPCR showed upregulation of cholesterol‐25‐hydroxylase (*Ch25h*) in cholesterol‐exposed CAFs (Figure 6J). Metabolomics analysis confirmed elevated production of 25‐HC, a CH25H‐catalyzed cholesterol metabolite (Figure 6K). RNA‐seq further identified CH25H as the essential enzyme mediating cholesterol‐induced myCAF differentiation, as siRNA knockdown abolished this effect (Figure 6L; S7I–K). Conversely, exogenous 25‐HC treatment in CAFs rescued *Ch25h*‐deficient myCAF conversion via RT‐qPCR (Figure S7L,M). GO enrichment analysis of upregulated DEGs in cholesterol‐exposed CAFs showed ECM remodeling and cytoskeletal rearrangement enrichment, whereas *Ch25h*‐deficient CAFs exhibited inverse enrichment patterns (Figure 6M). In vivo, shRNA‐mediated *Ch25h* knockdown reduced collagen deposition, myCAFs differentiation and matrix stiffness, and inhibited high‐cholesterol diet (HCD)‐treated CAFs to promote castration resistance in PCa (Serum cholesterol level of NCD: 2.36±0.12 mmol/L; HCD:3.72±0.45 mmol/L; Figure 6N, S7N‐P).

### 2.6 Blocking IRE1α‐XBP1s or Biosynthesis of Cholesterol Attenuates CRPC Progression

**FIGURE 7:**
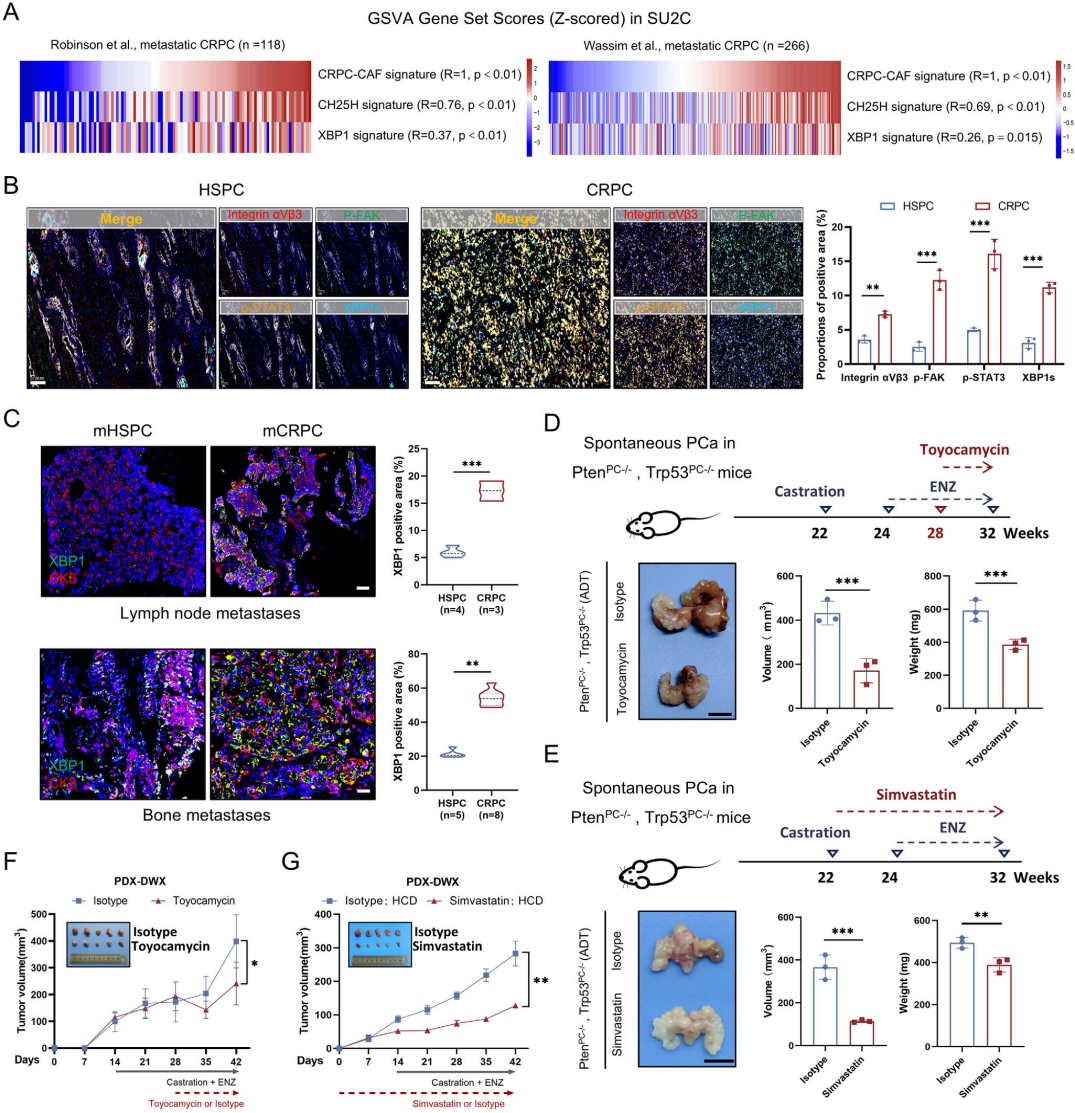
Blocking IRE1α‐XBP1s or biosynthesis of cholesterol attenuates CRPC progression. (A) Heatmap summary of the correlations of the CH25H signature and XBP1 signature in CRPC datasets. (B) Representative images and quantification of Integrin αVβ3 in HSPC and CRPC samples from primary tumors (n = 3). Scale bar, 100 µm. (C) Representative images and quantification of XBP1 in HSPC and CRPC samples from lymph node metastasis and bone metastasis. Scale bar, 100 µm. (D) Pten^PC‐/−^, Trp53^PC‐/−^ castrated mice treated with Toyocamycin or isotype (n = 3). Scale bar, 1 cm. (E) Pten^PC‐/−^, Trp53^PC−/−^ castrated mice treated with Simvastatin or isotype (n = 3). Scale bar, 1 cm. (F,G) Images of subcutaneous tumors and the growth curves of volume in HSPC PDX‐DWX mice treated with Toyocamycin or isotype and simvastatin or isotype (n = 5). *p < 0.05, **p < 0.01, ***p < 0.001.

To assess the clinical relevance of XBP1 signaling and CAF‐associated cholesterol metabolism in CRPC, we generated XBP1 and CH25H gene signatures to infer pathway activity. CH25H signature was derived from genes positively correlated with CH25H in TCGA PRAD, and that the XBP1 signature comprises core components of the integrin αVβ3/FAK/STAT3/XBP1 axis. Analysis of human datasets demonstrated a positive correlation between the XBP1/CH25H signature and CRPC‐CAF signature reported previously (Figure 7A). Multiplex IF staining further demonstrated robust activation of the integrin αVβ3/FAK/STAT3/XBP1s axis in CRPC tissues (Figure 7B), with significantly elevated XBP1s expression in lymph node and bone metastases, an effect exhibiting tissue‐type dependence (Figure 7C). To evaluate therapeutic potential, we investigated whether inhibition of IRE1α‐XBP1s signaling using Toyocamycin or cholesterol synthesis using simvastatin could suppress CRPC progression in Pten/Trp53 DKO mice and patient‐derived xenograft (PDX) models. Compared to ADT monotherapy, combination treatment with either Toyocamycin (Figure 7D, F) or simvastatin (Figure 7E, G) significantly reduced tumor volume and weight, confirming synergistic effects. In light of these findings, we propose that combining ADT with IRE1α‐XBP1s signaling inhibition or cholesterol synthesis blockade may represent a promising strategy to delay CRPC progression.

## 3 Discussion

Acquisition of resistance to ADT confers highly metastatic potential and worse clinical outcome in CRPC [3]. While matrix stiffness is an established hallmark of therapy resistance in solid tumors, its role in evolution of CRPC remains elusive. Here, we delineate a metabolic‐mechanotransductive crosstalk orchestrating CRPC progression. Integrating clinical cohort, genetically engineered mouse models, and tunable PDMS‐based culture systems, we establish matrix stiffness as a critical biomechanical determinant of therapeutic resistance. Mechanistically, ADT reprograms cholesterol metabolism in PCa cells, driving CAF differentiation and consequent ECM remodeling. This matrix stiffening sustains XBP1s‐dependent survival programs through mechotransduction, abrogating ADT‐induced apoptosis and fueling CRPC progression.

CAFs are established as pivotal regulators of matrix stiffness and therapeutic resistance. The lineage plasticity of CAF drives PCa progression, supported by scRNA and multi‐cohort histological validation [31, 32]. Consistent with prior scRNA‐seq in spontaneous PCa mouse model [8], our integrated analysis of human PCa datasets revealed significant myCAF enrichment in CRPC, implicating its critical role in therapeutic resistance. However, the biomechanical role of myCAFs in CRPC remains unclear. Our findings show that CD248 is highly enriched in CRPC‐specific myCAF subset and coincides with matrix stiffness, promoting therapeutic resistance independent of CAF paracrine signaling. Depletion of CD248^+^ CAFs in *CD248^CreERT2‐tdTomato‐DTR^* mice attenuated matrix stiffness and suppressed CRPC progression. Nevertheless, the mechanisms by which matrix stiffness accelerates castration resistance require further dissection.

The unfolded protein response (UPR) represents a conserved adaptive mechanism to preserve proteostatic integrity. In malignancies, however, persistent stress co‐opts downstream signaling not merely for restoration of homeostasis, but to buffer against apoptosis and fuel therapeutic resistance [33, 34]. Chen et al. further demonstrated the role of splicing factors in inducing aberrant splicing events in therapeutic resistance [35]. Here, we identify matrix stiffness as a critical extrinsic driver of CRPC progression through UPR activation. While matrix stiffness typically induces profound alterations in cell behaviors through diverse mechanosensitive receptors, ranging from surface sensors like integrins and discoidin domain receptor 1 (DDR1) to lipid‐gated ion channels like Piezo1 and TRPV4 [36, 37], our transcriptomic profiling identifies the UPR effector XBP1 as a central mediator PCa. Matrix stiffness engages the IRE1α‐XBP1s axis to confer resistance to FAD‐mediated apoptosis, directly linking stromal mechanics to cell survival [38]. A prominent finding is the elucidation of a mechanotransductive “bypass” mechanism sustaining tumor viability during ADT. Physiologically, AR signaling maintains UPR by binding enhancer of *IRE1α* and *XBP1u* [29], creating a feedback loop wherein XBP1s stabilizes AR and upregulates pro‐survival targets [39]. In CRPC, however, we show that matrix stiffness compensates for the loss of androgenic signaling by reactivating IRE1α‐XBP1s pathway via a dual mechanism: the direct stimulation of IRE1α RNase activity for XBP1 splicing, and the concurrent activation of integrin αVβ3/FAK/STAT3 axis to transcriptionally replenish *XBP1u* pools. This coordinated input ensures sustained substrate supply and enzymatic activity, amplifying IRE1α‐XBP1s signaling to evade apoptosis under therapeutic stress. Consequently, disruption of the IRE1α‐XBP1s axis, either through direct IRE1α kinase inhibition or upstream STAT3 blockade, effectively attenuates CRPC progression in vivo.

Beyond targeting mechanotransduction, inhibiting the differentiation of CAFs into myCAFs represents a complementary therapeutic approach to reduce matrix stiffness and suppress CRPC progression. Consequently, therapeutic strategies disrupting TME‐CAF crosstalk have emerged as promising strategies to inhibit cancer progression [40]. In PCa, AR inhibition activates TGF‐β/Smad/SOX4 axis by restoring TGFβR1 promoter activity, promoting CAF‐to‐myCAF transition through SWI/SNF complex‐dependent chromatin remodeling that is suppressed by TGF‐β inhibition [8]. Metabolites further orchestrate CAF phenotypic plasticity. For instance, glutamine limitation in PDAC maintains the myCAF phenotype via macropinocytosis‐mediated suppression of inflammatory reprogramming, highlighting metabolic adaption mechanisms [41]. Our work reveals that androgen deprivation enhances cholesterol synthesis and secretion in PCa cells. Although cholesterol metabolism has been implicated in CRPC progression [42, 43], its role in CAF phenotypic modulation remains undefined. Here, we demonstrate that the exogenous cholesterol derived PCa cells drives CH25H‐dependent phenotypic conversion of CAFs to myCAFs. Genetic ablation of *Ch25h* effectively attenuates myCAF differentiation and matrix stiffness, ultimately delaying castration resistance. These findings establish the cholesterol‐CH25H as a central metabolic driver of stromal reprogramming in CRPC.

There are also some limitations in our study. Our data support stiffness‐associated restoration of IRE1α‐XBP1 signaling under FAD, while the precise upstream mechanism may involve stiffness‐induced proteostatic stress and remains to be further refined. Additionally, our study validated Integrin αVβ3 as the primary mechanosensory and detected abnormal elevations in several other intermediate cholesterol metabolites. The other mechanosensitive receptors and the exact biological functions of other metabolites have yet to be fully defined, and the potential contributions to this process cannot be completely excluded. For animal models and clinical cohort, potential differences between murine models and human CRPC need further to be accessed. Therefore, the therapeutic approaches with Toyocamycin and simvastatin as preclinical evidence supports translational potential, rather than broad clinical generalization and immediate clinical applicability, limited by the modest size of our human cohort. Besides, androgen deprivation therapy was not completely uniform, and that such heterogeneity may influence stromal remodeling and biomechanical states.

## 4 Conclusion

In summary, we elucidate a cholesterol‐driven metabolic‐mechanotransductive crosstalk in CRPC, wherein CH25H‐dependent stromal reprogramming promotes castration resistance through matrix stiffening and pro‐survival mechanosignaling. This work provides a compelling therapeutic rationale for co‐targeting cholesterol metabolism or mechanosignaling alongside ARSIs to disrupt the feed‐forward loop driving CRPC progression.

## 5 Experimental Section

### 5.1 Human Specimens

Patient samples were collected at Xijing Hospital. The use of pathological specimens and pathological information was approved by the patients' informed consent and the Ethics Committee of Xijing Hospital. The patient samples involved included 8 pairs of paired in situ PCa samples from HSPC to CRPC (Table S3), primary PCa samples from 19 HSPC and 21 CRPC patients, lymph node metastasis samples from 4 mHSPC and 3 mCRPC patients, and bone metastasis samples from 5 mHSPC and 8 mCRPC patients (Table S4). The recurrent CRPC tissues were obtained from patients who had received androgen‐directed therapy, including antiandrogens such as apalutamide and enzalutamide, before sampling. All metastases were identified histologically by Department of Pathology of Xijing Hospital. It should be specifically noted that surgery is not the primary treatment for CRPC, and CRPC samples were obtained from palliative surgery or pathological puncture.

### 5.2 Animals

All mice were maintained in specific pathogen‐free (SPF) facilities. All relevant protocols were conducted in accordance with the guidelines for the care and use of laboratory animals and were approved by the Ethics Committee of Xijing Hospital and the Laboratory Animal Center of the Fourth Military Medical University. Pbsn‐Cre and Trp53‐floxed transgenic mice were purchased from Cyagen Biosciences, and Pten‐floxed transgenic mice were purchased from Shanghai Model Organisms. Cd248^CreERT2^ and Rosa26^LSL‐DTR‐tdTomato^ transgenic mice were generated by Shanghai Model Organisms using CRISPR/Cas9 gene editing technology. All genetically engineered mouse models were maintained in C57BL/6 background.

For animal model construction and treatment, ADT was used as combination of castration and enzalutamide (ENZ) administration (25 mg/kg, three times per week; i.p.). For Pten^PC‐/−^; Trp53^PC‐/−^ mice, spontaneous prostate cancer development occurred at variable times; therefore, mice were monitored by MRI at 22–28 weeks of age. Castration was performed 1 month following PCa onset, with ENZ administration initiated 2 weeks post‐surgery and continued for 8 weeks. For the subcutaneous tumor‐bearing model, 5 × 10^6^ tumor cells were resuspended in 50 µL Matrigel and inoculated subcutaneously into the dorsal region of mice. Castration was performed 1 week after tumor inoculation, and ENZ administration was initiated 1 week post‐surgery and continued for 4 weeks. For in vivo modulation of matrix stiffness, tumor site skin area was measured following establishment, and 0.5 mg/mL type I collagenase (250 µL/cm^2^, twice weekly) was injected peritumorally until sacrifice, as the protocol described [27]. For CD248^CreERT2‐tdTomato‐DTR^ mice, 1 × 10^6^ DKO cells were orthotopically injected to induce tumor formation. 1 week after initiating ENZ treatment, tamoxifen was administered (120 mg/kg, every other day for 5 doses; i.p.), followed by diphtheria toxin (DT; 50 µg/kg, every other day; i.v.) until sacrifice. For IgG78 antibody treatment, mice received injections (10 mg/kg; i.v.) for 4 weeks prior to sacrifice. For mixed tumor inoculation, 3 × 10^6^ Myc‐Cap or 22RV1 cells mixed with 1 × 10^6^ CAFs were co‐injected into dorsal of 8‐week‐old FVB or nude mice subcutaneously. For PDX models, CRPC tissues were dissected into 2–3 mm^3^ tissue fragments and implanted into 6‐week‐old male NSG mice. ADT was maintained via combined castration and ENZ administration. In vivo administration of drugs including Stattic (MCE, HY‐13818), Toyocamycin (MCE, HY‐103248) and simvastatin (MCE, HY‐17502) was performed per manufacturer instructions. Subcutaneous tumor xenograft models and PDX models were established in 6 – 8‐week‐old male C57BL/6, FVB, Tramp, nude, and NSG mice. Tumor volume was measured every 7 days using vernier calipers using volume formula: 0.5 × (length × width^2^). The volume of orthotopic spontaneous tumors was observed by small animal MRI and analyzed using ITK‐SNAP software.

### 5.3 Cell Lines and Organoids Models

Cell lines were purchased from the American Type Culture Collection (ATCC), Thermo Fisher Scientific, and the Cell Bank of the Chinese Academy of Sciences' Typical Culture Preservation Committee (National Collection of Authenticated Cell Cultures), and were cryopreserved in liquid nitrogen in our laboratory. Myc‐Cap, Tramp‐C1, 22RV1, and 293T cells were cultured in DMEM or 1640 medium containing 10% FBS at 37°C with 5% CO_2_. 293F cells were cultured in suspension using FreeStyle 293‐F Expression Medium on a cell shaker at 37°C, 8% CO_2_, and 125 rpm/min. Cell lines were tested to confirm the absence of mycoplasma contamination. EPCAM^−^CD31^−^CD45^−^ cells were considered cancer‐associated fibroblasts (CAFs) and were derived from PCa tissues of human or FVB mice. Pten; Trp53 DKO cells were derived from PTEN/Trp53 DKO mice.

Tumor tissue was minced into 1–3 mm^3^ blocks in a sterile biosafety cabinet. Digestion solution was added at a 5:1 volume ratio versus tissue, followed by incubation at 37°C for 30 min. Upon microscopic observation of abundant cell clusters, primary culture buffer was added to terminate digestion. The mixture was filtered through a 100‐µm cell sieve and centrifugation was performed at 300×g for 5 min at 4°C, and the supernatant was discarded. Cell clusters were resuspended in melted Matrigel (Matrigel:cell pellet volume ratio = 25:1) and thoroughly mixed. A 25 µL aliquot of the Matrigel‐cell cluster mixture was added to each well of a 24‐well plate, which was then placed in a 37°C cell incubator till the Matrigel was solidified. Organoid culture medium (500 µL) was added for subsequent cultivation, with medium changes every 24 h. Subculturing was performed when organoids reached a diameter of 200–500 µm. Organoid growth was monitored using a spinning‐disk confocal microscope, images were acquired, and analyses were performed using Image J software. Organoids were cultured according to previously reported methods [44]. A 25‐µL mixture of Matrigel and cell clumps (Matrigel volume: cell clump pellet volume = 25:1) was added to each well of a 24‐well plate. The growth of organoids was observed using a spinning disk confocal microscope. Passage operations were performed when the organoids reached a diameter of 200–500 µm.

### 5.4 Preparation of PDMS Matrices

PDMS substrates were fabricated by mixing dimethylsiloxane monomer with a cross‐linking agent at defined ratios, as previously described [45]. For cell culture, substrates with distinct mechanical stiffness were prepared by adjusting the monomer:cross‐linking agent ratio. Briefly, the two components were vigorously mixed to ensure homogeneity, then allowed to stand at room temperature for 1 h to eliminate air bubbles. The degassed mixture was poured into 6‐well plates (200 µL per well) and cured in an oven at 65°C for at least 4 h to form a solid PDMS layer. A 0.2% dopamine solution was freshly prepared in 10 mM Tris‐HCl buffer (pH 8.5) prior to use. Prior to cell seeding, 500 µL of the dopamine solution was added to each well to functionalize the PDMS surface. The plates were incubated in a humidified cell culture incubator (37°C, 5% CO_2_) for 2 h, retrieved, and washed three times with sterile 1×PBS to remove unbound dopamine.

### 5.5 Integrated Analysis of scRNA‐seq Data

Single‐cell RNA sequencing datasets of human prostate cancer were integrated using R. Dataset metadata, including accessions, sample characteristics, and reference, is listed in Table S1. The *Harmony* R package was used to correct batch effects across datasets. Cell clustering was performed based on the expression of canonical cell surface markers. The differentiation trajectory of CAFs was reconstructed through pseudotime analysis.

### 5.6 Immunostaining and Immunohistochemistry (IHC)

Tissues were fixed with 4% paraformaldehyde, embedded in paraffin, and sectioned at 6 µm. After dewaxing and hydration, antigen retrieval was performed in citrate buffer. Sections were incubated with an endogenous peroxidase blocker, followed by blocking with goat serum. Subsequently, sections were incubated with primary antibody overnight at 4°C, then with secondary antibodies for 1 h at room temperature. Antibody and reagents information and RRIDs are provided in the Table S5. For IF, sections were mounted with antifade mounting medium containing DAPI. For IHC, followed with DAB solution, sections were differentiated, blued, dehydrated, cleared, and mounted. Sections were scanned using a digital slide scanner, and at least 3 random sections per tissue source were selected for statistical analysis.

For cytoskeleton immunofluorescence staining, a PDMS cell culture substrate was prepared in a confocal dish. Tumor cells were digested and counted, with 1 × 10^3^ cells seeded onto the PDMS; complete medium was added to a final volume of 1 mL, and the dish was transferred to a cell incubator for 48 h of culture at 37°C with 5% CO_2_. Cells were fixed with pre‐cooled paraformaldehyde for 30 min, followed by washes with PBS. Then permeabilization was sequentially performed with 0.1% Triton X‐100 for 5 min and iFlour 647‐phalloidin working solution (100 µL per dish) at room temperature (RT) in the dark. DAPI‐containing anti‐fade mounting medium was then added, with a 5‐min incubation at RT in the dark. Finally, cell morphology was imaged using a spinning‐disk confocal microscope. Cell area was quantified with Image J software, and statistical analysis was performed using GraphPad Prism.

### 5.7 Collagen, Tissue Morphology and Stiffness Assessment

For decellularization assay, sterilized cell slides were coated by 2% gelatin solution and transferred to a 37°C cell incubator for 1 h, followed by rinse with DPBS. Then the slides were sequentially incubated in 1% glutaraldehyde solution and 1 M isobutanol solution at room temperature for 30 min, respectively. CAFs were plated onto the cell slides and incubated with complete medium for 24 h. The medium was changed to complete medium supplemented with 50 µg/mL ascorbic acid, with medium changes every 24 h. After 7–9 days of conventional culture, the slides were removed and sequentially incubated with 1 mL cell lysis buffer for 10 min and DNase for 30 min (prewarmed to 37°C), followed by rinse with DPBS. For paraffin section, preparation and dewaxing followed standard histological staining protocols. Cell slides and sections were imaged using a two‐photon microscope, and collagen fiber parameters (length, width, orientation) were quantified. Data were statistically analyzed using MATLAB, Image J and GraphPad Prism software. Coefficient of variation (CV) was calculated as: CV = (Standard deviation/Mean) ×100%.

For morphology assessment, paraffin section preparation and dewaxing followed standard immunohistochemical staining procedures. For stiffness detection, fresh tissues were sequentially dehydrated in 15% and 30% sucrose solutions, snap‐frozen in liquid nitrogen, and embedded in OCT compound. Embedded tissues were stored at −80°C until sectioning. Prior to assessment, tissues were cut into 80 µm slices using a cryostat. Samples were analyzed for stiffness via atomic force microscopy (AFM), and data were processed using MATLAB, R, and GraphPad Prism.

### 5.8 Tissue Hydroxyproline Content Quantification

Approximately 0.2 g of tissue was weighed into an EP tube and minced. 2 mL hydrochloric acid extraction solution was added, and tissues were digested in a 105°C metal bath until no large clumps remained. Hydroxyproline content detection kit (Solarbio, BC0250) was used for tissue hydroxyproline content quantification. A standard curve was generated by plotting standard concentration (x‐axis) against ΔA (y‐axis), yielding the equation *y* = *kx*+*b*. ΔA values from measurement tubes were substituted into this equation to calculate *x* (µg/mL). Tissue hydroxyproline content (µg/g) was determined using the formula: hydroxyproline content = (x × *V*_sample_)/ (W × *V*_sample_/*V*_tissue extract_) × F. (W: weight of samples; F: dilution multiple)

### 5.9 Isolation of Primary CAFs, Cell Culture and Treatment

PCa tissues were digested until blocks were completely dispersed, and the resulting cell suspension was filtered through a 70‐µm pore size filter to generate a single‐cell suspension. Red blood cells were subsequently removed from the tissue using lysis buffer. Details can be found in previously reported experimental protocols [8, 46].CD31, EPCAM, and CD45 antibodies were added sequentially to the cell suspension, and triple‐negative cells sorted by flow cytometry were identified as CAFs. The cells were washed with PBS and resuspended in DMEM medium supplemented with 20% fetal bovine serum, then cultured in a 37°C incubator with 5% CO_2_. For sorting CD248^+^ CAFs, cells were incubated with 100 nM CD248 antibody followed by flow cytometric sorting. Antibody information and RRIDs are listed in the Table S5. For cholesterol treatment, 0.75 µg/ml cholesterol (with or without 0.5 mM β‐CD) was added to the CAF medium, and cells were collected for protein or mRNA detection after 48 h of culture.

PCa cell lines were cultured in DMEM/RPMI‐1640 medium supplemented with 10% fetal bovine serum. For full androgen deprivation, DMEM/RPMI‐1640 medium containing 10% charcoal‐stripped serum was used, and ENZ was added at concentrations of 5 µM for 22RV1 cells or 20 µM for Myc‐Cap and Tramp‐C1 cells according to their half maximal inhibitory concentration (IC50). The medium was changed every two days, and cells were cultured for 7 days for proliferation assessment via cell counting or crystal violet staining. Drugs and reagents were used according to the manufacturer's instructions; reagent information and sources are provided in the Table S5. For conditioned medium collection, tumor cells were allowed to grow to 80% confluence before the culture medium was harvested. The medium was centrifuged at 2000 g/min for 10 min to remove cellular debris, then filtered through a 0.22‐µm sterile filter. The resulting filtrate was collected and stored at −80°C. For experimental applications, the conditioned medium was mixed with complete medium supplemented with 10% FBS at a 1:1 ratio. CAFs were cultured in this mixture for 48 h prior to the initiation of subsequent assays.

*RT‐qPCR*: For the isolation of total RNA from tissues and cells, TRIzol reagent (YEASEN, Shanghai, China) was used. A 1 µg aliquot of total RNA was reverse‐transcribed into cDNA using the Hifair III first Strand cDNA Synthesis SuperMix for qPCR (YEASEN, cat. no. 11141ES), following the manufacturer's protocol. Real‐time quantitative PCR (qPCR) was performed on a Bio‐Rad iCycler iQ system (Bio‐Rad, Hercules, CA, USA) using the Hifair qPCR SYBR Green Master Mix (YEASEN, cat. no. 11201ES). Primer sequences are provided in Table S6. Relative gene expression levels were calculated using the 2^−ΔΔCt^ method.

### 5.10 Immunoblotting

Cells were lysed with RIPA lysis buffer, and proteins were separated by SDS‐PAGE followed by transfer onto a polyvinylidene difluoride (PVDF) membrane. The membrane was incubated with primary antibodies overnight at 4°C, followed by a 1‐h room temperature incubation with horseradish peroxidase‐conjugated anti‐rabbit/mouse secondary antibodies. The membrane was washed with TBST, and ECL luminescent solution was used for signal detection on the CHAMPCHEMI Chemiluminescent Imaging System (Sagecreation, Beijing, China). Antibody information and RRIDs are provided in the Table S5, and all antibodies were used at the dilution ratios recommended by the manufacturer's instructions.

### 5.11 Flow Cytometry for Apoptosis Detection

Cells in the culture supernatant and adherent cells were collected by digestion and centrifugation. Samples were centrifuged at 300×g/min for 5 min at 4°C, and the supernatant was discarded. Cells were washed with pre‐cooled PBS, followed by staining using Annexin V/PI apoptosis detection kit (Bestbio, BB‐4101). Prior to sample loading, quality control samples were prepared from the test cells to establish fluorescence compensation. Following fluorescence compensation adjustment, routine flow cytometry procedures were performed.

### 5.12 Collagen Gel Contraction Assay

CAFs were digested and counted. A total of 5 × 10^4^ cells/well were mixed with 1 mg/mL type I collagen and 3 mg/mL Matrigel, and 0.5 mL/well of the cell‐type I collagen‐Matrigel mixture was added to a 96‐well plate. After incubation at 37°C for 1 h, the gel was released, and 1 mL of cell culture medium containing 2% serum was added to the collagen gel. The 96‐well plate was transferred to a cell culture incubator for 48 h of culture. Subsequently, gel size was observed and measured under a gel imaging system at 48 h, and images were analyzed using Image J software.

### 5.13 Expression and Purification of Antibody

293F suspension cells were cultured in a shaker at 37°C, 8% CO_2_, and 135 rpm/min. The IgG78 plasmid was transfected into cells using FreeStyle MAX transfection reagent (Gibco, 16447100), with cell number, reagent volume, and transfection steps performed according to the manufacturer's instructions. Cell supernatant was collected after 7 days of culture and filtered under negative pressure through a 0.45‐µm pore‐size membrane. A Protein A chromatography column was mounted on the protein purification system; following column regeneration and equilibration, cell supernatant, binding buffer, and elution buffer were passed through the column sequentially, and antibodies bound to the resin were collected. Antibodies were neutralized with 1 m Tris‐HCl buffer and dialyzed against PBS. Sterilization was performed via filtration through a 0.22‐µm membrane, and antibody concentration was quantified using NanoDrop platform. Antibodies were stored at ‐80°C.

### 5.14 Plasmid and Cloning

*Stat3* overexpression plasmid and *Ch25h*‐siRNA was synthesized by Generalbiol, CO., LTD (Anhui, China). Sense and anti‐sense KD oligonucleotides targeting *Ch25h, Cps1, Igfbp5, Sidt2, Tmem176b, Cxcl17, Xbp1, Retreg3, Gata2, Rest, Foxa1, Stat3, Ptk2* were annealed and cloned into pLKO.1‐puro vector (Addgene). The shRNA and siRNA sequences are provided in Table S6.

### 5.15 Infections and Transfections

HEK293T cells were used for virus amplification. pMD2G and psPAX2 plasmids were utilized for lentiviral packaging of shRNA, with PEI reagent employed for plasmid transfection. Empty vectors served as controls for both shRNA‐mediated knockdown and overexpression experiments. For the florescence labeling for CAFs and organoids in the co‐culture system, mCherry and EGFP plasmids were utilized for lentiviral packaging, with PEI reagent employed for plasmid transfection. Culture supernatant collected at 48 and 72 h was filtered through a 0.45‐µm pore size filter, followed by centrifugation, mixing, and storage at −80°C. For siRNA and dual luciferase reporter gene plasmid transfection, Lipofectamine 3000 (Invitrogen) was used to transiently deliver plasmid into cells. Medium was changed 6 h post lentiviral infection or transfection, and cell lines were selected using puromycin 48 h later.

### 5.16 DNA Pull‐Down

A biotin‐labeled Xbp1 promoter probe was constructed based on the Xbp1 promoter sequence in the pUC57 vector. DNA amplification was used to enhance probe yield, followed by purification of the labeled probe using a gel recovery kit and quantification. Probe primer sequences are provided in Table S6. After immobilizing the DNA probe on streptavidin magnetic beads, the protein sample was co‐incubated with the probe. Proteins bound to the DNA probe were eluted with an eluent, and those interacting with XBP1 were detected and identified via mass spectrometry and immunoblotting.

### 5.17 ChIP‐qPCR

Chromatin immunoprecipitation experiments were carried out in PCa cells using the ChIP kit (JKR23002A, Gene Create). Chromatin from 1 × 10^7^ cells was used for each ChIP reaction with p‐STAT3 antibody (9145, Cell Signaling Technology). Purified DNA was prepared for qPCR. The primer sequences are provided in Table S6.

### 5.18 Bulk RNA‐Seq Analysis

TRIzol reagent (YEASEN, Shanghai, China) was used for the isolation of total RNA from cells. Each group contained RNA from 3 independent biological replicates. The RNA libraries were subjected to paired‐end 150 (PE150) Hiseq and sequenced on the illumina Novaseq 6000 platform by LC Bio Technology CO.,Ltd (Hangzhou, China). The list of significance was operated by setting fold changes threshold at level of 1.5 and *p* < 0.05 using DEseq2. The raw data can be downloaded as PRJNA1347950 and PRJNA1347867 from SRA database.

### 5.19 ATAC‐Seq and Data Analysis

1 × 10^6^ CAFs were lysed and subjected to ATAC‐seq, with sequencing performed by Wuhan Igenebook Biotechnology Co., Ltd. Each group included samples from two independent biological replicates. The quality of purified libraries was accessed using Bioanalyzer and Q‐bit. ATAC‐seq libraries were then sequenced on the Illumina NovaSeq 6000 platform with PE150 method. Raw data were quality‐controlled using FastQC (v. 0.11.5), followed by adapter trimming and filtering with Trimmomatic (v. 0.36). Clean reads were aligned to the GRCm39 reference genome using HISAT2 (v. 2.0.1‐beta). Read distribution around gene transcription start sites (TSSs; ±2 kb) was analyzed for aligned BAM files using deepTools (v. 2.5.4), with an initial significance threshold set at a q‐value of 0.05. Peak region read counts were quantified via DiffBind (v. 1.16.3), and differential accessibility analysis was performed between groups. The raw data can be downloaded as PRJNA1347950 from SRA database.

### 5.20 Metabolomics and Proteomics

Steroids contents were detected by MetWare (http://www.metware.cn/) based on the AB Sciex QTRAP 6500 LC‐MS/MS platform. Each group contained 3 independent biological replicates. The sample extracts were analyzed using an LC‐ECI‐MS/MS system (UPLC, ExionLC AD, https://sciex.com.cn/; MS, QTRAP 6500+ System, https://sciex.com /). AB 6500+ QTRAP LC‐MS/MS System was equipped with an ESI Turbo Ion‐Spray interface, operating in positive ion mode and controlled by Analyst 1.6 software (AB Sciex). Significantly regulated metabolites between groups were determined by absolute Log_2_FC (fold change; ≧2 and ≦0.5).

Protein samples were analyzed by nano‐liquid chromatography‐tandem mass spectrometry (nano‐LC‐MS/MS) using an UltiMate 3000 RSLCnano system coupled with a Q Exactive HF mass spectrometer (Thermo) in data‐dependent acquisition (DDA) mode. Raw data were processed with MaxQuant software (v. 2.2.0.0) using the Andromeda database search algorithm. Spectra files were searched against the Mouse protein sequence database (UniProt, 3 January 2023). Results were filtered to a 1% false discovery rate (FDR). Bioinformatics analyses were performed in R. Functional annotations were performed using GO, KEGG, EggNOG, Pfam, and UniProt subcellular localization databases.

### 5.21 Analysis of CRPC‐CAF, CH25H, XBP1 Signature in Human CRPC

The CRPC‐CAF signature was derived as previously described [8]. To construct the CH25H signature, Pearson correlation coefficients between Ch25h and other genes were calculated using the TCGA PRAD dataset. The signature was defined by the top 148 genes exhibiting significant positive correlation with CH25H (r > 0.5, *p* < 0.05). The XBP1 signature comprises *Itga5*, *Itgb3*, *Ptk2*, *Stat3*, and *Xbp1*, the key components of the Integrin αVβ3/FAK/STAT3/XBP1 signaling axis. Gene Set Variation Analysis (GSVA) was employed to generate enrichment scores for each signature, where higher scores indicate coordinated upregulation of the constituent genes within the respective signature.

### 5.22 Statistical Analysis

Statistical analysis was performed using GraphPad Prism 9 (GraphPad Software, Inc.), with data presented as mean ± SEM from at least three independent experiments. Assumptions for parametric tests were assessed based on standard evaluation of data distribution and variance in GraphPad Prism. For comparisons between two independent groups, two‐tailed paired or unpaired t‐tests were used. For comparisons among three or more groups, one‐way or two‐way ANOVA was performed, followed by multiple comparisons. Survival was analyzed using the Kaplan‐Meier method with univariate analysis. All in vitro experiments were independently repeated at least three times, and in vivo experiments included at least five independent biological replicates with consistent time courses and treatments. Statistical significance was defined as *p* < 0.05, where * denotes *p* < 0.05, **p < 0.01, and ***p < 0.001.

## Author Contributions

**S. Liu**: Conceptualization, data curation, investigation, methodology, writing – original draft. **C. Xu**: Visualization, formal analysis. **J. Jiang**: Data curation, funding acquisition. **L. He**: Software. **Y. Zhou**: Resources. **Z. Li**: Resources. **Y. Li**: Funding acquisition. **K. Zhang**: Visualization. **F. Yang**: Resources. **T. Lu**: Methodology. **H. Song**: Investigation. **H. Zhu**: Software. **Z. Hu**: Visualization. **X. Zhao**: Validation. **K. Gan**: Validation. **H. Li**: Writing – review and editing. **B. Yang**: Writing – review and editing. **R. Zhang**: Project administration. **W. Wen**: Project administration. **D. Han**: Funding acquisition. **W. Qin**: Conceptualization, Supervision, funding acquisition.

## Conflicts of Interest

The authors declare no conflict of interest.

## Supporting information




**Supporting File 1**: advs75977‐sup‐0001‐SuppMat.docx.

**Supporting File 2**: advs75977‐sup‐0002‐FigureS1‐S7.zip.

## Data Availability

The data that support the findings of this study are openly available in SRA database at https://dataview.ncbi.nlm.nih.gov/, reference number PRJNA1347867 and PRJNA1347950.
