# Supplementary material for: Cholesterol‐Mediated Metabolic‐mechanotransductive Crosstalk Orchestrates Castration Resistance in Prostate Cancer
Source: Adv Sci (Weinh). 2026 Jun 4;13(48):e75977. doi: 10.1002/advs.75977 (PMC13336987; doi:10.1002/advs.75977)
Supplement: Supplementary file 1 [file ADVS-13-e75977-s002.docx]

**Supplemental information**

**Cholesterol-mediated** **metabolic-mechanotransductive crosstalk orchestrates castration resistance in prostate cancer**

Shaojie Liu, Chao Xu, Jun Jiang, Limin He, Yike Zhou, Zhengxuan Li, Yu Li, Keying Zhang, Fa Yang, Tong Lu, Hongtao Song, Hai Zhu, Zhihao Hu, Xiaolong Zhao, Kai Gan, Hongji Li, Bo Yang, Rui Zhang, Weihong Wen, Donghui Han, Weijun Qin

**Figure S1.** **Matrix stiffness increases in CRPC murine models, related to Figure 1.**

(A) Schematic of spontaneous and castration-resistant prostate cancer murine model establishment.
(B) Representative images and quantification of Masson staining for collagen, immunohistochemical (IHC) staining for p-Paxillin and p-MLC2, and immunofluorescence (IF) staining for AR. Scale bar, 100μm.
(C) Representative SHG imaging via two-photon microscopy revealing deep collagen architecture, with quantitative analysis of fiber density and alignment.
(D) Schematic of tumor-Associated Collagen Signature (TACS), TACS scoring and statistical comparison between groups.
(E) Pearson correlation analysis between TACS scores and tumor volumes.
(F) Hydroxyproline quantification assay demonstrating collagen content in fresh tumor tissues.
(G) AFM topographical profiling of tumor surface morphology.
(H) AFM-based mapping of Young's modulus distribution across tumor regions.

n(sham)=9, n(ADT)=8. *P<0.05, **P<0.01, ***P<0.001.

**Figure S2.** **Bioinformatics analysis profiles the PCa stromal heterogeneity, related to Figure 1.**

(A) Representative AFM topographical profiling of tumor surface morphology.
(B) Biomarkers of distinct cell subpopulations.
(C) UMAP visualization of re-clustered fibroblasts populations with overlaid marker expression profiles.
(D) Tissue preference of each cluster, in which the Ro/e denotes the ratio of observed over expected cell numbers.
(E) Top 5 differentially expressed genes (DEGs) defining fibroblast subsets.
(F) Estimation of stromal and immune cells in HSPC and CRPC using transcriptome data from GSE70769 and GSE32269.
(G) Kaplan-Meier survival analysis for StromalScore based on prognostic data from TCGA.

**Figure S3. Matrix stiffness promotes the progression of CRPC, related to Figure 2.**

(A) Representative images and statistical analysis of Masson and SHG imaging of PCa tissues (n=3). Scale bar, 100 μm.
(B) Detection of hydroxyproline content in PCa tissues (n=3).
(C) Statistical analysis of the elastic modulus of PCa tissues detected by AFM (n=3, 10 points per sample).
(D) Schematic diagram of the construction of a flow cytometry sorting and co-culture system.
(E) Representative images and statistical analysis of the number and size of Myc-Cap cells clones cultured on the soft and stiff matrices in the co-culture system (n=3).
(F) Proliferation curves of PCa cells on the soft and stiff matrices in the co-culture system with changes in time (n=3).

ns，no significance. *P＜0.05，** P＜0.01，***P＜0.001.

**Figure S4.** **CD248^+^ CAFs promote ECM secretion and collagen contraction, related to Figure 3.**

(A) Expression of CD248 in five subgroups of CAFs analyzed by single-cell sequencing.

(B) CD248 expression in CAFs detected by Immunoblotting.

(C) Representative images and statistical analysis of immunofluorescence staining for Ki-67 in DKO organoids (n=3). Scale bar, 20μm.

(D) Representative images and statistical analysis of immunofluorescence staining for spontaneous fluorescence of orthotopic transplanted tumors in CD248Cre^ERT-tdTomato-DTR^ mice (n = 3). Scale bar, 100μm.

(E) Growth curve of the volume of orthotopic transplanted tumors in CD248Cre^ERT-tdTomato-DTR^ mice detected by MRI (n=5).

(F-H) Representative images (F) and statistical analysis of Masson staining (G) and SHG imaging (H) of orthotopic transplanted tumors from CD248Cre^ERT-tdTomato-DTR^ mice (n=5). Scale bar, 100μm.

(I) Hydroxyproline quantification assay demonstrating collagen content of orthotopic transplanted tumors from CD248Cre^ERT-tdTomato-DTR^ mice (n=5).

(J-K) Representative images (J) and statistical analysis (K) of the tissue morphology and Young's modulus of orthotopic transplanted tumors from CD248Cre^ERT-tdTomato-DTR^ mice detected by AFM (n=5).

** P＜0.01，***P＜0.001.

**Figure S5. Matrix stiffness sustains PCa cell survival under FAD treatment through activation of IRE1α-XBP1s signaling pathway, related to Figure 4.**

(A-B) Transcription levels of AR target genes (A) and NEPC markers (B) in Myc-Cap cells detected by RT-qPCR (n=3).

(C) PCA of Myc-Cap cells in the soft and stiff groups (n=3).

(D) Expression levels of the indicated genes in Myc-Cap cells after shRNA knockdown detected by RT-qPCR (n=3).

(E) Statistical analysis of the growth of Myc-Cap cells after Toyocamycin blocking the IRE1α-XBP1s signaling (n=3).

(F) GSEA analysis of the KEGG terms of "response to endoplasmic reticulum stress".

(G) Expression levels of Xbp1u and Xbp1s in Myc-Cap cells detected by RT-qPCR (n=3).

(H) Expression of the principle genes of IRE1α-XBP1s signaling pathway in Myc-Cap cells detected by Immunoblotting.

(I) RT-qPCR analysis of Eif2ak3/Elf2, Atf6 and Ern1 expression in Myc-Cap cells on the soft and stiff substrates under FAD treatment. (n=3).

(J-K) Expression levels of Xbp1 in Myc-Cap cells infected with shRNA and overexpression plasmids detected by RT-qPCR (J; n=3) and Immunoblotting (K).

(L) Representative images of flow cytometry detection of apoptosis in Myc-Cap cells with FAD treatment.

(M) Survival analysis of subcutaneous tumor bearing FVB mice treated with ADT (n=5).

ns, no significance; **P<0.01, ***P<0.001.

**Figure S6.** **Matrix stiffness promotes XBP1 expression via Integrin αVβ3/FAK/STAT3 signaling pathway, related to Figure 5.**

(A) Expression levels of the indicated genes in Myc-Cap cells after shRNA knockdown detected by RT-qPCR (n=3).

(B) Immunoblotting assay detects the expression levels of STAT3 and XBP1 in PCa cells.

(C) Joint analysis of the binding peak of STAT3 in the Xbp1 promoter region by Cistrome DB and UCSC Browser websites.

(D) Silver staining image of proteins that directly interact with the Xbp1 probe in the DNA pull-down experiment.

(E) Prediction of the binding site of STAT3 to the Xbp1 promoter by the JASPAR website.

(F) Motif sequence diagram of the binding site of STAT3 and Xbp1 promoter in human cells.

(G) Immunoblotting assay detects the expression level of STAT3 in PCa cells stably transfected by overexpression plasmid.

(H) Dual-luciferase reporter gene assay detects the luciferase activity of the Xbp1 promoter sites that binds to STAT3 in 293T cells (n=3).

(I) ChIP-qPCR detects the sequence amplification of the Xbp1 promoter sites that binds to STAT3 in 293 cells (n=3).

(J) Survival rate of Myc-Cap cells treated with gradient concentrations of Stattic via CCK8 assay (n=3).

ns, no significance; ***P<0.001.

**Figure S7. CH25H-mediated cholesterol metabolic rewiring in CRPC drives CAFs polarization, related to Figure 6**

(A) Heatmaps comparing the scaled normalized expression of cholesterol synthases in WT or FAD-treated Myc-Cap cells on soft substrates.

(B) GSEA analysis of the DEGs in the “Cholesterol biosynthetic process” .

(C) RT-qPCR analysis of the myCAF signature gen es expression in CAFs cultured alone or co-cultured with CRPC (n=3).

(D) Representative IF images of Filipin III MFI in CAFs cultured alone or with CRPC conditioned medium (n=3). Scale bar, 20μm。

(E) RT-qPCR analysis of the myCAF signature genes expression in CAFs incubated with gradient concentrations of cholesterol (n=3).

(F) Immunoblotting analysis of myCAFs markers expression in CAFs.

(G) Representative SHG images of ECM content and structure organization secreted by CAFs with or without cholesterol treatment in cell slides, with quantification (n=3). Scale bar, 20 μm

(H) Representative images of collagen gel contraction assay demonstrating matrix remodeling mediated by CAFs with or without cholesterol treatment, with quantitative analysis (n=5).

(I) PCA of the transcriptome across the CAF subsets.

(J) Volcano plot of the DEGs across the CAF subsets.

(K) Immunoblotting validation of CH25H expression and analysis of myCAFs markers expression in CAFs.

(L) Immunoblotting validation of CH25H expression in cholesterol-treated CAFs.

(M) RT-qPCR analysis of the myCAF signature genes and Ch25h expression in CAFs incubated with cholesterol (n=3).

(N) Representative images and quantification of Masson staining for collagen and IHC staining for α-SMA (n=3). Scale bar, 100μm.

(O) Detection of hydroxyproline content in PCa tissues (n=3).

(P) Statistical analysis of the elastic modulus of PCa tissues detected by AFM (n=3, 10 points per sample).

*P<0.05, **P<0.01, ***P<0.001.

**Table S1. PCa datasets of single-cell RNA sequence data, related to Figure 1**

| NO. | Cell count | Source data | Sample | Ref |
| --- | --- | --- | --- | --- |
| 1 | 179,359 | Gene Expression Omnibus database (accession code: GSE181294) | Normal: 20  Primary PCa: 19 | 19 |
| 2 | 57,000 | Gene Expression Omnibus database (accession code: GSE185344) | Normal: 7  Primary PCa: 7 | 20 |
| 3 | 21,743 | Gene Expression Omnibus database (accession code: GSE176031) | Normal: 4  Primary PCa: 11 | 21 |
| 4 | 16,529 | Gene Expression Omnibus database (accession code: GSE193337) | Primary PCa: 4 | 22 |
| 5 | 21,292 | Gene Expression Omnibus database (accession code: GSE137829) | CRPC: 6 | 23 |
| 6 | 24,385 | SRA database (accession code: PRJNA699369) | CRPC: 2  mCRPC: 1 | 24 |
| 7 | 36,424 | Gene Expression Omnibus database (accession code: GSE141445) | Normal: 6  HSPC: 12  CRPC: 5 | 5 |
| 8 | 27,338 | Gene Expression Omnibus database (accession code: GSE210358) | CRPC: 6 | 25 |
| 9 | 7,904 | Gene Expression Omnibus database (accession code: GSE157703) | HSPC: 2 | 26 |

**Table S2. ChIP-seq database screening results used to nominate upstream transcription factors of Xbp1，related to Figure 5**

| hTFtarget | KnockTF | ENCODE | GTRD | ChIP_Atlas |
| --- | --- | --- | --- | --- |
| SPI1 | TP53 | ARID3A | AFF1 | ACTR5 |
| ATF2 | FOXA1 | ATF1 | AFF4 | AFF1 |
| BACH2 | ARID1A | ATF2 | AGO2 | AFF4 |
| BATF | HOXA1 | ATF3 | AHR | AHR |
| BCL11A | TCF3 | BACH1 | APP | AKAP8 |
| BHLHE40 | FLI1 | BATF | AR | AR |
| BRD4 | ZIC2 | BCLAF1 | ARID1A | ARID1A |
| CBFB | TP63 | BHLHE40 | ARID2 | ARID1B |
| CDK7 | LIN28A | BRCA1 | ARNT | ARID2 |
| CDK8 | TAL1 | CCNT2 | ARNTL | ARID4A |
| CDK9 | HIF1A | CEBPB | ARRB1 | ARID4B |
| CEBPA | FOXP1 | CEBPD | ASCL1 | ARID5B |
| CEBPB | MITF | CHD1 | ASH2L | ARNT |
| CREB1 | YBX1 | CHD2 | ATF1 | ASCL1 |
| CREBBP | IRF4 | CHD7 | ATF2 | ASCL2 |
| CTCF | SALL4 | CREB1 | ATF3 | ATF1 |
| E2F1 | ZIC5 | CTCF | ATXN7L3 | ATF2 |
| E2F4 | ARNT | CUX1 | BACH1 | ATF3 |
| EBF1 | ELK3 | E2F4 | BCL11A | ATF4 |
| ELF1 | REST | E2F6 | BCL11B | ATXN7L3 |
| EP300 | ETV1 | EBF1 | BCL3 | BAHCC1 |
| ERG | CBFB | ELF1 | BCL6 | BANF1 |
| FLI1 | SOX2 | ELK1 | BCOR | BANP |
| FOS | HOXD9 | ELK4 | BHLHE40 | BCL11B |
| FOXM1 | POLR2G | EP300 | BICRA | BCL3 |
| GATA1 | RBM17 | ETS1 | BMI1 | BCOR |
| GATA2 | SAFB2 | EZH2 | BRCA1 | BCORL1 |
| GATA3 | FIP1L1 | FOS | BRD2 | BHLHE40 |
| HDAC1 | PCBP1 | FOSL2 | BRD3 | BMI1 |
| HDAC2 | TARDBP | FOXA1 | BRD4 | BRD2 |
| IRF1 | SRSF3 | FOXA2 | BRD7 | BRD3 |
| IRF4 | PRPF4 | FOXM1 | BRD9 | BRD4 |
| JUND | PCBP2 | FOXP2 | BRPF3 | BRD7 |
| KLF1 | DPF2 | GABPA | c-myc | BRD9 |
| LMO2 | ILF3 | GATA1 | CAT | BRPF3 |
| MAX | RBM39 | GATA2 | CBX1 | CBX3 |
| MAZ | NFATC1 | GATA3 | CCND2 | CCNT2 |
| MED1 | SMAD5 | GTF2B | CCNT2 | CDK6 |
| MEF2A | TFDP1 | GTF2F1 | CDK12 | CDK7 |
| MTA3 | HSF1 | GTF3C2 | CDK7 | CDK8 |
| MYB | MAF1 | H2AFZ | CDK8 | CDK9 |
| MYC | RBM15 | HCFC1 | CDK9 | CDKN1B |
| MYH11 | RBM22 | HDAC1 | CDKN1B | CDYL2 |
| NFIC | AR | HDAC2 | CDX2 | CEBPA |
| NFYB | FOXO1 | HDAC6 | CEBPA | CEBPB |
| PAX5 | GATA2 | HMGN3 | CEBPB | CEBPD |
| PBX3 | GATA6 | HNF4G | CEBPD | CEBPG |
| POLR2A | HES6 | IKZF1 | CENPA | CHD1 |
| POU2F1 | HNF4a | IRF1 | CHD1 | CHD6 |
| POU2F2 | HTATIP | IRF3 | CHD2 | CHD8 |
| RAD21 | HTATIP2 | JUN | CHD8 | CIC |
| RARA | Klf4 | JUND | CLOCK | CNOT3 |
| REST | KLF5 | KAT2A | CNOT3 | CPSF2 |
| RFX5 | MEF2D | KDM4A | CREB1 | CREB1 |
| RUNX1 | MYB | KDM5A | CREB3L1 | CREB3L2 |
| RUNX1T1 | PHB | KDM5B | CREBBP | CREBBP |
| RUNX3 | SOX4 | MAFK | CREM | CREM |
| SP1 | STAT3 | MAX | CRTC2 | CRTC2 |
| STAG1 | TBX3 | MAZ | CSNK2A1 | CTCF |
| STAT1 | TET1 | MBD4 | CTBP1 | CXXC5 |
| TAF1 | TET2 | MEF2A | CTCF | DAXX |
| TAL1 | ZHX2 | MTA3 | CTCFL | DDIT3 |
| TBP | MARK2 | MXI1 | CXXC1 | DEAF1 |
| TBX21 | NKRF | MYBL2 | CXXC4 | DLX5 |
| TCF12 | XRCC5 | MYC | DCP1A | DLX6 |
| TCF3 | FUBP1 | MYOD1 | DDX20 | DMAP1 |
| TCF7L2 |  | MYOG | DEAF1 | DOT1L |
| TFAP4 |  | NELFE | DLX1 | DR1 |
| VDR |  | NFATC1 | DMAP1 | DRAP1 |
| YY1 |  | NFIC | DMC1 | DYRK1A |
| ETS1 |  | NFYA | DNMT3B | DZIP1 |
| ATF3 |  | NFYB | DOT1L | E2F1 |
| E2F6 |  | NR2F2 | DPF2 | E2F4 |
| KDM5B |  | NRF1 | DRAP1 | E2F5 |
| KMT2A |  | PAX5 | E2F1 | EEA1 |
| NFYA |  | PHF8 | E2F4 | EED |
| PBX1 |  | PML | E2F6 | EGR1 |
| PHF8 |  | POLR2A | E2F8 | ELF1 |
| RBBP5 |  | POU2F2 | EBF1 | ELL2 |
| SIRT6 |  | RAD21 | EGR3 | EP300 |
| THAP1 |  | RBBP5 | ELF1 | EP400 |
| USF1 |  | RCOR1 | ELK3 | ERCC3 |
| ZBTB7A |  | RELA | ELL2 | ERG |
| ZNF384 |  | REST | EMSY | ESR1 |
| GABPA |  | RFX5 | EP300 | ETS1 |
| SIN3A |  | RUNX3 | EP400 | ETV1 |
| ZFX |  | RXRA | EPAS1 | ETV6 |
| AHR |  | SAP30 | ERCC2 | FLI1 |
| AR |  | SETDB1 | ERCC3 | FOS |
| ARNT |  | SIN3A | ERCC6 | FOSL2 |
| BRCA1 |  | SIRT6 | ERG | FOXA1 |
| EGLN2 |  | SMC3 | ESR1 | FOXA2 |
| ESR1 |  | SP1 | ETS1 | FOXO1 |
| FOXA1 |  | SP2 | ETV1 | FOXP4 |
| GRHL2 |  | SP4 | ETV2 | GABPA |
| HIF1A |  | SPI1 | ETV5 | GABPB1 |
| JUN |  | SRF | EWSR1 | GATA1 |
| KLF4 |  | STAT1 | EZH2 | GATA2 |
| NR2F2 |  | STAT3 | FANCD2 | GATA3 |
| PGR |  | STAT5A | FEZF1 | GATA4 |
| RARG |  | SUZ12 | FGFR1 | GLIS1 |
| SRC |  | TAF1 | FIP1L1 | GLIS2 |
| STAT3 |  | TAF7 | FLI1 | GMEB1 |
| TFAP2C |  | TAL1 | FOS | GPN1 |
| TP53 |  | TBL1XR1 | FOSL2 | GRHL2 |
| XBP1 |  | TBP | FOXA1 | GRHL3 |
| FOXA2 |  | TCF12 | FOXA2 | GTF2B |
| ELL2 |  | TCF3 | FOXA3 | GTF2E2 |
| GTF2B |  | TCF7L2 | FOXM1 | GTF2F1 |
| HCFC1 |  | TEAD4 | FOXO1 | HDAC1 |
| SFMBT1 |  | THAP1 | FOXO3 | HDAC2 |
| UBN1 |  | TRIM28 | FOXP1 | HES4 |
| BCL6 |  | UBTF | FUS | HEXIM1 |
| E2F8 |  | USF1 | GABPA | HIF1A |
| ETS2 |  | USF2 | GABPB1 | HIVEP1 |
| ETV5 |  | WHSC1 | GATA1 | HMGN3 |
| GMEB2 |  | WRNIP1 | GATA2 | HMGXB4 |
| HOXA6 |  | YY1 | GATA3 | HNF4A |
| MED12 |  | ZBTB7A | GATA4 | HNRNPC |
| NFYC |  | ZKSCAN1 | GATA6 | HNRNPK |
| NIPBL |  | ZMIZ1 | GATAD2A | HNRNPLL |
| RBCK1 |  | ZNF143 | GATAD2B | HOXA3 |
| TAF3 |  | ZNF263 | GLIS1 | HOXA4 |
| BCOR |  | ZNF384 | GLIS2 | HOXA6 |
| CHD1 |  |  | GMEB2 | HOXB13 |
| FOXP1 |  |  | GRHL2 | HSF1 |
| OTX2 |  |  | GRHL3 | IKZF1 |
| SMAD4 |  |  | GTF2B | IKZF3 |
| TAF7 |  |  | GTF2F1 | IKZF4 |
| TEAD4 |  |  | H2AFZ | ING2 |
| PPARG |  |  | HCFC1 | ING5 |
| GTF2I |  |  | HDAC1 | INTS10 |
| MAFB |  |  | HDAC2 | INTS12 |
| ETV1 |  |  | HDAC4 | INTS13 |
| CEBPD |  |  | HDGF | INTS3 |
| WDR5 |  |  | HDGFL3 | INTS5 |
| BRD2 |  |  | HEXIM1 | INTS6 |
| SUMO2 |  |  | HEY1 | ISL2 |
| TBL1X |  |  | HIC1 | JADE3 |
| TRIM28 |  |  | HIF1A | JUN |
| ZNF263 |  |  | HIF3A | JUND |
| ARID3A |  |  | HIRA | KAT6A |
| EZH1 |  |  | HMGA1 | KAT6B |
| HNF4A |  |  | HMGXB4 | KAT7 |
| HNF4G |  |  | HNF1B | KDM1A |
| MBD4 |  |  | HNF4A | KDM2A |
| MXI1 |  |  | HNF4G | KDM2B |
| MYBL2 |  |  | HNRNPC | KDM4A |
| NFE2 |  |  | HNRNPH1 | KDM4C |
| RXRA |  |  | HNRNPK | KDM5B |
| SMC3 |  |  | HNRNPL | KDM6A |
| USF2 |  |  | HNRNPLL | KLF1 |
| ZEB1 |  |  | HOMEZ | KLF11 |
| LMNB1 |  |  | HOXA6 | KLF12 |
| RBL2 |  |  | HOXB13 | KLF17 |
| RELA |  |  | HOXC9 | KLF3 |
| MYOD1 |  |  | HSF1 | KLF5 |
| KDM4C |  |  | IKZF1 | KLF7 |
| DDX5 |  |  | IKZF2 | KLF8 |
| DUX4 |  |  | IKZF3 | KMT2A |
| KLF9 |  |  | IKZF5 | KMT2B |
| NRIP1 |  |  | INTS11 | L3MBTL2 |
| ONECUT1 |  |  | INTS12 | LARP7 |
| SMARCA4 |  |  | INTS13 | LBX2 |
| SP2 |  |  | INTS3 | LIN54 |
| KLF5 |  |  | IRF1 | LIN9 |
| ARRB1 |  |  | IRF3 | MAFB |
| CBX8 |  |  | IRF4 | MAX |
| NR3C1 |  |  | IVNS1ABP | MAZ |
| RUNX2 |  |  | JMJD6 | MBD3 |
| SRF |  |  | JUN | MEAF6 |
| ELK3 |  |  | JUNB | MED1 |
|  |  |  | JUND | MED12 |
|  |  |  | KAT7 | MED13 |
|  |  |  | KDM1A | MED26 |
|  |  |  | KDM2B | MEF2B |
|  |  |  | KDM4A | MEIS2 |
|  |  |  | KDM4C | MEN1 |
|  |  |  | KDM5B | MGMT |
|  |  |  | KDM5C | MITF |
|  |  |  | KDM6A | MLLT1 |
|  |  |  | KDM6B | MLLT6 |
|  |  |  | KLF11 | MNT |
|  |  |  | KLF12 | MNX1 |
|  |  |  | KLF16 | MTOR |
|  |  |  | KLF4 | MXD1 |
|  |  |  | KLF5 | MXD3 |
|  |  |  | KLF6 | MXI1 |
|  |  |  | KLF9 | MYB |
|  |  |  | KMT2A | MYC |
|  |  |  | KMT2B | MYCN |
|  |  |  | L3MBTL2 | MYOCD |
|  |  |  | L3MBTL4 | MYOD1 |
|  |  |  | LDB1 | MZF1 |
|  |  |  | LEF1 | N6AMT1 |
|  |  |  | LEO1 | NABP2 |
|  |  |  | LIN9 | NAIF1 |
|  |  |  | LMO1 | NCAPH2 |
|  |  |  | MAF | NCBP1 |
|  |  |  | MAFB | NELFA |
|  |  |  | MAX | NELFB |
|  |  |  | MAZ | NELFCD |
|  |  |  | MBD2 | NELFE |
|  |  |  | MBD3 | NEUROD1 |
|  |  |  | MBD4 | NFIA |
|  |  |  | MBL2 | NFYA |
|  |  |  | ME3 | NFYB |
|  |  |  | MECP2 | NFYC |
|  |  |  | MED12 | NIPBL |
|  |  |  | MED26 | NONO |
|  |  |  | MEIS3P1 | NR0B2 |
|  |  |  | MEN1 | NR1H3 |
|  |  |  | MITF | NR2C2 |
|  |  |  | MIXL1 | NR2F1 |
|  |  |  | MLLT1 | NR2F2 |
|  |  |  | MLX | NR3C1 |
|  |  |  | MNT | NRF1 |
|  |  |  | MORC2 | NRL |
|  |  |  | MTA2 | NUTM1 |
|  |  |  | MTA3 | OGT |
|  |  |  | MUC22 | PAF1 |
|  |  |  | MXD3 | PATZ1 |
|  |  |  | MXI1 | PAX5 |
|  |  |  | MYB | PAX8 |
|  |  |  | MYBL2 | PBRM1 |
|  |  |  | MYC | PBX3 |
|  |  |  | MYCN | PBX4 |
|  |  |  | MYOD1 | PCBP1 |
|  |  |  | MYOG | PGR |
|  |  |  | NANOG | PHF2 |
|  |  |  | NCAPH2 | PHF21A |
|  |  |  | NCOA1 | PHF8 |
|  |  |  | NCOA3 | POGZ |
|  |  |  | NCOA4 | POLR2C |
|  |  |  | NCOR1 | POU5F1 |
|  |  |  | NELFA | PPARG |
|  |  |  | NELFE | PRDM10 |
|  |  |  | NEUROD1 | PROSER1 |
|  |  |  | NFATC1 | PRPF4 |
|  |  |  | NFE2 | RAD21 |
|  |  |  | NFE2L2 | RAG2 |
|  |  |  | NFIA | RARA |
|  |  |  | NFIC | RB1 |
|  |  |  | NFIL3 | RBBP5 |
|  |  |  | NFKB1 | RBBP7 |
|  |  |  | NFKB2 | RBFOX2 |
|  |  |  | NFKBIZ | RBL1 |
|  |  |  | NFYA | RBM22 |
|  |  |  | NFYB | RBM25 |
|  |  |  | NFYC | RBPJ |
|  |  |  | NIPBL | REL |
|  |  |  | NKX2-1 | RELA |
|  |  |  | NONO | REST |
|  |  |  | NOTCH1 | RFX5 |
|  |  |  | NR1H2 | RFXANK |
|  |  |  | NR1H3 | RFXAP |
|  |  |  | NR2F1 | RORC |
|  |  |  | NR2F2 | RUNX1 |
|  |  |  | NR2F6 | RUNX1T1 |
|  |  |  | NR3C1 | RUNX2 |
|  |  |  | NR5A2 | RUVBL2 |
|  |  |  | NRF1 | RXRA |
|  |  |  | NUP98 | RXRB |
|  |  |  | OGG1 | SAP130 |
|  |  |  | OLIG2 | SETD1A |
|  |  |  | ONECUT2 | SIN3A |
|  |  |  | OR2M7 | SKI |
|  |  |  | OTX2 | SMAD2 |
|  |  |  | OVOL2 | SMAD3 |
|  |  |  | p65 | SMAD4 |
|  |  |  | PALB2 | SMAD5 |
|  |  |  | PATZ1 | SMARCA2 |
|  |  |  | PAX3 | SMARCA4 |
|  |  |  | PAX5 | SMARCB1 |
|  |  |  | PCBP1 | SMARCC1 |
|  |  |  | PCF11 | SMC1A |
|  |  |  | PDX1 | SNAI2 |
|  |  |  | PEX2 | SNAPC1 |
|  |  |  | PGR | SP1 |
|  |  |  | PHF2 | SP2 |
|  |  |  | PHF5A | SP7 |
|  |  |  | PHF8 | SPEN |
|  |  |  | PITX3 | SPI1 |
|  |  |  | PML | SREBF2 |
|  |  |  | POU2F1 | SS18 |
|  |  |  | POU2F2 | SS18L1 |
|  |  |  | POU5F1 | STAG1 |
|  |  |  | PPARG | STAG2 |
|  |  |  | PRMT1 | STAT1 |
|  |  |  | PRPF4 | STAT2 |
|  |  |  | PTBP1 | STAT3 |
|  |  |  | PTEN | STAT5A |
|  |  |  | RAD21 | SUMO2 |
|  |  |  | RAG1 | SUPT5H |
|  |  |  | RAG2 | SUPT6H |
|  |  |  | RARA | SUZ12 |
|  |  |  | RARG | TAF1 |
|  |  |  | RB1 | TAF7 |
|  |  |  | RBBP4 | TAF9B |
|  |  |  | RBBP5 | TBP |
|  |  |  | RBFOX2 | TBPL1 |
|  |  |  | RBL2 | TCF25 |
|  |  |  | RBM14 | TCF7L2 |
|  |  |  | RBM15 | TET2 |
|  |  |  | RBM22 | TFAP2A |
|  |  |  | RBM25 | TFAP2C |
|  |  |  | RBPJ | TFAP4 |
|  |  |  | RCOR2 | TFDP1 |
|  |  |  | REL | TFDP2 |
|  |  |  | RELA | THAP12 |
|  |  |  | RELB | TIGD6 |
|  |  |  | REST | TOX4 |
|  |  |  | RFX1 | TP53 |
|  |  |  | RFX5 | TP53BP1 |
|  |  |  | RFXANK | TRIM28 |
|  |  |  | RNF2 | TWIST1 |
|  |  |  | RORC | USF1 |
|  |  |  | RUNX1 | USP7 |
|  |  |  | RUNX1T1 | VCP |
|  |  |  | RXRA | VDR |
|  |  |  | RXRB | VSX2 |
|  |  |  | SAP130 | WAS |
|  |  |  | SAP30 | WDR5 |
|  |  |  | SETD7 | WDR77 |
|  |  |  | SETDB1 | WT1 |
|  |  |  | SFPQ | XBP1 |
|  |  |  | SIN3A | YEATS4 |
|  |  |  | SIRT6 | YWHAZ |
|  |  |  | SKI | YY1 |
|  |  |  | SLC30A9 | ZBTB20 |
|  |  |  | SMAD1 | ZBTB21 |
|  |  |  | SMAD2 | ZBTB26 |
|  |  |  | SMAD3 | ZBTB38 |
|  |  |  | SMAD4 | ZBTB48 |
|  |  |  | SMAD5 | ZBTB7A |
|  |  |  | SMARCA2 | ZFP82 |
|  |  |  | SMARCA4 | ZFP91 |
|  |  |  | SMARCB1 | ZFX |
|  |  |  | SMARCC1 | ZFY |
|  |  |  | SMC1A | ZGPAT |
|  |  |  | SMC3 | ZHX2 |
|  |  |  | SNAI2 | ZMAT3 |
|  |  |  | SNRNP70 | ZMYM3 |
|  |  |  | SOX13 | ZMYND8 |
|  |  |  | SOX2 | ZNF219 |
|  |  |  | SOX4 | ZNF225 |
|  |  |  | SOX9 | ZNF230 |
|  |  |  | SP1 | ZNF232 |
|  |  |  | SP140 | ZNF280B |
|  |  |  | SP2 | ZNF329 |
|  |  |  | SP4 | ZNF333 |
|  |  |  | SP5 | ZNF335 |
|  |  |  | SPI1 | ZNF346 |
|  |  |  | SREBF2 | ZNF350 |
|  |  |  | SRF | ZNF394 |
|  |  |  | SRSF1 | ZNF430 |
|  |  |  | SRSF3 | ZNF431 |
|  |  |  | SRSF4 | ZNF441 |
|  |  |  | SRSF7 | ZNF446 |
|  |  |  | SSRP1 | ZNF449 |
|  |  |  | SSU72 | ZNF451 |
|  |  |  | STAT1 | ZNF460 |
|  |  |  | STAT3 | ZNF485 |
|  |  |  | STAT5A | ZNF501 |
|  |  |  | STAT5B | ZNF543 |
|  |  |  | SUMO2 | ZNF547 |
|  |  |  | SUPT5H | ZNF574 |
|  |  |  | SUPT6H | ZNF598 |
|  |  |  | SUZ12 | ZNF605 |
|  |  |  | T-Cell | ZNF607 |
|  |  |  | TAF1 | ZNF616 |
|  |  |  | TAF3 | ZNF619 |
|  |  |  | TAF7 | ZNF629 |
|  |  |  | TAL1 | ZNF660 |
|  |  |  | TARDBP | ZNF691 |
|  |  |  | TBL1XR1 | ZNF692 |
|  |  |  | TBP | ZNF703 |
|  |  |  | TBX21 | ZNF709 |
|  |  |  | TCF12 | ZNF710 |
|  |  |  | TCF3 | ZNF737 |
|  |  |  | TCF7L1 | ZNF770 |
|  |  |  | TCF7L2 | ZNF772 |
|  |  |  | TEAD1 | ZNF773 |
|  |  |  | TEAD3 | ZNF782 |
|  |  |  | TEAD4 | ZNF839 |
|  |  |  | TET2 | ZNF865 |
|  |  |  | TET3 | ZNF883 |
|  |  |  | TFAP2A | ZNF891 |
|  |  |  | TFAP2C | ZSCAN30 |
|  |  |  | TFAP4 | ZSCAN31 |
|  |  |  | TFDP1 | ZXDC |
|  |  |  | TFE3 |  |
|  |  |  | THAP11 |  |
|  |  |  | THRB |  |
|  |  |  | TP53 |  |
|  |  |  | TP53BP1 |  |
|  |  |  | TP63 |  |
|  |  |  | TRIM24 |  |
|  |  |  | TRIM25 |  |
|  |  |  | TRIM28 |  |
|  |  |  | TRPS1 |  |
|  |  |  | U2AF1 |  |
|  |  |  | UBTF |  |
|  |  |  | USF1 |  |
|  |  |  | USF2 |  |
|  |  |  | USP7 |  |
|  |  |  | VDR |  |
|  |  |  | WDR5 |  |
|  |  |  | WT1 |  |
|  |  |  | XBP1 |  |
|  |  |  | XRCC5 |  |
|  |  |  | YAP1 |  |
|  |  |  | YY1 |  |
|  |  |  | YY2 |  |
|  |  |  | ZBTB14 |  |
|  |  |  | ZBTB17 |  |
|  |  |  | ZBTB25 |  |
|  |  |  | ZBTB26 |  |
|  |  |  | ZBTB33 |  |
|  |  |  | ZBTB40 |  |
|  |  |  | ZBTB48 |  |
|  |  |  | ZBTB6 |  |
|  |  |  | ZBTB7A |  |
|  |  |  | ZC3H8 |  |
|  |  |  | ZEB1 |  |
|  |  |  | ZEB2 |  |
|  |  |  | ZFP42 |  |
|  |  |  | ZFP64 |  |
|  |  |  | ZFX |  |
|  |  |  | ZHX2 |  |
|  |  |  | ZIC2 |  |
|  |  |  | ZIC5 |  |
|  |  |  | ZKSCAN1 |  |
|  |  |  | ZMIZ1 |  |
|  |  |  | ZMYM3 |  |
|  |  |  | ZMYND8 |  |
|  |  |  | ZNF143 |  |
|  |  |  | ZNF148 |  |
|  |  |  | ZNF207 |  |
|  |  |  | ZNF224 |  |
|  |  |  | ZNF263 |  |
|  |  |  | ZNF3 |  |
|  |  |  | ZNF35 |  |
|  |  |  | ZNF350 |  |
|  |  |  | ZNF366 |  |
|  |  |  | ZNF48 |  |
|  |  |  | ZNF511 |  |
|  |  |  | ZNF574 |  |
|  |  |  | ZNF618 |  |
|  |  |  | ZNF639 |  |
|  |  |  | ZNF770 |  |
|  |  |  | ZSCAN22 |  |
|  |  |  | ZSCAN29 |  |
|  |  |  | ZSCAN9 |  |

**Table S3. Paired sample information for human PCa patients, related to Figure 1**

| NO. | Age | Date of diagnosis | Gleason score | PFS | Gleason score |
| --- | --- | --- | --- | --- | --- |
| 1 | 88 | 2/2021 | 8 | 24 months | 8 |
| 2 | 73 | 12/2019 | 9 | 10 months | 9 |
| 3 | 62 | 9/2019 | 7 | 11 months | 7 |
| 4 | 63 | 7/2019 | 9 | 9 months | 9 |
| 5 | 58 | 5/2018 | 8 | 23 months | 9 |
| 6 | 65 | 5/2016 | 9 | 25 months | 9 |
| 7 | 63 | 10/2016 | 9 | 42 months | 9 |
| 8 | 83 | 1/2010 | 9 | 65 months | 9 |

**Table S4. Clinical summary of the HSPC and CRPC patients, related to Figure 7.**

| Vatiables | Primary prostate | | Lymph node metastasis | | | Bone metastasis | | |
| --- | --- | --- | --- | --- | --- | --- | --- | --- |
|  | HSPC patients | CRPC patients | | HSPC patients | CRPC patients | | HSPC patients | CRPC patients |
| Numbers | 19 | 21 | | 4 | 3 | | 5 | 8 |
| Age at diagnosis | 54-87 | 60-88 | | 44-66 | 66-78 | | 55-81 | 58-66 |
| Pathologic Gleason score in primary prostate tumor at initial diagnosis, n (%) | | | | | | | | |
| 6 | 0（0） | 1（4.8） | | 0（0） | 0 | | 0 | 0 |
| 7 | 7（36.8） | 2（9.5） | | 1（25.0） | 1（33.3） | | 0 | 0 |
| 8 | 6（31.6） | 7（33.3） | | 2（50.0） | 0 | | 1（20.0） | 0 |
| 9 | 6（31.6） | 11（52.4） | | 1（25.0） | 1（33.3） | | 4（80.0） | 5（62.5） |
| 10 | 0（0） | 0（0） | | 0（0） | 1（33.3） | | 0 | 3（37.5） |

**Table S5. The key reagents and resource**

| Reagent or Resource | Source | Identifier |
| --- | --- | --- |
| Antibodies |  |  |
| p-Paxillin | Cell Signaling Technology | Cat. #69363; RRID: AB_3095333 |
| p-MLC2 | Cell Signaling Technology | Cat. #95777; RRID: AB_3677547 |
| CK8 | Proteintech | Cat. #10384; RRID: AB_3083875 |
| AR | Proteintech | Cat. #22089; RRID: AB_11182176 |
| EPCAM | Biolegend | Cat. #369809; RRID: AB_2650906 |
| EPCAM | Biolegend | Cat. #118213; RRID: AB_1134105 |
| CD31 | Biolegend | Cat. #102417; RRID: AB_830756 |
| CD31 | Biolegend | Cat. #303117; RRID: AB_2114314 |
| CD45 | Biolegend | Cat. #982306; RRID: AB_2650649 |
| CD45 | Biolegend | Cat. #103125; RRID: AB_493536 |
| CD248 | Abcam | Cat. #ab67273; RRID: AB_1143398 |
| COL1A1 | Cell Signaling Technology | Cat. #72026; RRID: AB_2904565 |
| α-SMA | Proteintech | Cat. #14395-1; RRID: AB_2223009 |
| CH25H | Santa Cruz Biotechnology | Cat. #sc-293256; RRID: AB_3076545 |
| NOTCH1 | Proteintech | Cat. #20687-1; RRID: AB_10700012 |
| XBP1s | Proteintech | Cat. #24868; RRID: AB_2879766 |
| XBP1u | Proteintech | Cat. #25997; RRID: AB_2880326 |
| IRE1α | Cell Signaling Technology | Cat. #3294; RRID: AB_823545 |
| p-IRE1α | Abcam | Cat. #ab48187; RRID: AB_873899 |
| BIP | Cell Signaling Technology | Cat. #3177; RRID: AB_2119845 |
| CHOP | Cell Signaling Technology | Cat. #2895; RRID: AB_2089254 |
| GAPDH | Proteintech | Cat. #60004; RRID: AB_2920461 |
| β-actin | Proteintech | Cat. #66009; RRID: AB_2919581 |
| p-STAT3 | Cell Signaling Technology | Cat. #9145; RRID: AB_2491009 |
| STAT3 | Cell Signaling Technology | Cat. #4904; RRID: AB_331269 |
| STAT3 | Proteintech | Cat. #60199; RRID: AB_2919659 |
| Integrin αⅤβ3 | Santa Cruz Biotechnology | Cat. # sc-7312; RRID: AB_627819 |
| FAK | Proteintech | Cat. #12636; RRID: AB_2173668 |
| p-FAK | Cell Signaling Technology | Cat. #3283; RRID: AB_2173659 |
| Dylight®488 Donkey anti-Rabbit | Abcam | Cat. #ab96919; RRID: AB_10679362 |
| Dylight®594 Donkey anti-Mouse | Abcam | Cat. #ab96877; RRID: AB_10679532 |
| Dylight®488 Goat anti-Human IgG Fc | Abcam | Cat. #ab97003; RRID: AB_10679804 |
| HRP conjugated Goat anti-Rabbit IgG | Abcam | Cat. #ab6721; RRID: AB_955447 |
| HRP conjugated Goat anti-Mouse IgG | Abcam | Cat. #ab205719; RRID: AB_2755049 |
| Chemicals |  |  |
| Masson staining solution | Servicebio | Cat. #G1006 |
| DAPI | Beyotime | Cat. #P0131 |
| PDMS substrates | Dowcorning | Cat. #DC-184 |
| Phalloidin | Yeasen | Cat. #40762ES75 |
| Filipin III | Absin | Cat. #abs42018484 |
| Isobutanol | Aladdin | Cat. #78-83-1 |
| Glutaraldehyde | Aladdin | Cat. #G105606 |
| Ascorbic acid | Aladdin | Cat. #A103539 |
| Gelatin | Sigma-Aldrich | Cat. #9000-70-8 |
| Tamoxifen | MedChem Express | Cat. #HY-13757A |
| Diphtheria Toxin | Sigma-Aldrich | Cat. #D0564 |
| Fetal Bovine Serum | Gibco | Cat. #10099141 |
| FreeStyle™ 293 Expression Medium | Gibco | Cat. #12338018 |
| OptiPRO™ SFM | Gibco | Cat. #12309050 |
| EZ Trans cell transfection reagent | Life-iLab | Cat. #AC04L092 |
| Cell lysate | Beyotime | Cat. #P0013 |
| Ammonium oxalate crystal violet staining solution | Biosharp | Cat. #BL802A |
| Collagenase I | Biosharp | Cat. #1904 |
| Collagenase IV | Biosharp | Cat. #2091 |
| T4 DNA ligase | Yeasen | Cat. #2011A |
| PEI transfection reagent | Yeasen | Cat. #40820ES |
| Cholesterol | MedChem Express | Cat. #HY-N0322 |
| 25-Hydroxycholesterol | MedChem Express | Cat. #HY-113134 |
| β-Cyclodextrin | MedChem Express | Cat. #HY-107201 |
| FreeStyle™ MAX Reagent | Gibco | Cat. #16447100 |
| Enzalutamide | MedChem Express | Cat. #HY-70002 |
| Toyocamycin | MedChem Express | Cat. #HY-103248 |
| Stattic | MedChem Express | Cat. #HY-13818 |
| IXA6 | MedChem Express | Cat. #HY-139212 |
| MKC8866 | MedChem Express | Cat. #HY-104040 |
| Simvastatin | MedChem Express | Cat. #HY-17502 |
| Critical commercial assays |  |  |
| Ready to use Immunohistochemistry Elivision™ plus kit | MXB Biotechnologies | Cat. #KIT-9922 |
| Annexin V/PI apoptosis detection kit | Bestbio | Cat. #BB-4101 |
| ChIP Kit | Gene Create | Cat. #JKR23002A |
| Hydroxyproline content detection kit | Solarbio | Cat. #BC0250 |
| Total cholesterol content detection kit | Solarbio | Cat. #BC1980 |
| Plasmid extraction kit | TIANGEN | Cat. #DP103 |
| Agorose gel DNA recovery kit | TIANGEN | Cat. #DP219-02 |

**Table S6. Oligonucleotides**

| Primers for RT-qPCR | Sequence (5’ to 3’) |
| --- | --- |
| mCd248-Forward | ACCAGATCCCCAAAATCAAGGC |
| mCd248-Reverse | GAGTGGCCGAGGTTATCCC |
| mAbca1-Forward | AAAACCGCAGACATCCTTCAG |
| mAbca1-Reverse | CATACCGAAACTCGTTCACCC |
| mAbcg1-Forward | CTTTCCTACTCTGTACCCGAGG |
| mAbcg1-Reverse | CGGGGCATTCCATTGATAAGG |
| mAcat1-Forward | GAAACCGGCTGTCAAAATCTGG |
| mAcat1-Reverse | TGTGACCATTTCTGTATGTGTCC |
| mAcat2-Forward | ACAAGACAGACCTCTTCCCTC |
| mAcat2-Reverse | ATGGTTCGGAAATGTTCACC |
| mLdlr-Forward | TGACTCAGACGAACAAGGCTG |
| mLdlr-Reverse | ATCTAGGCAATCTCGGTCTCC |
| mNpc1-Forward | GATATTGCCGGTCTTCCAGAG |
| mNpc1-Reverse | CTGGGCACTTTTTGCTTGATG |
| mNpc2-Forward | AGGACTGCGGCTCTAAGGT |
| mNpc2-Reverse | AGGCTCAGGAATAGGGAAGGG |
| mCh25h-Forward | TGCTACAACGGTTCGGAGC |
| mCh25h-Reverse | AGAAGCCCACGTAAGTGATGAT |
| mCyp27a1-Forward | CCAGGCACAGGAGAGTACG |
| mCyp27a1-Reverse | GGGCAAGTGCAGCACATAG |
| mCyp46a1-Forward | AGCCGCTATGAGCACATCC |
| mCyp46a1-Reverse | CCATACTTCTTAGCCCAATCCAG |
| mCyp7b1-Forward | GCCTTTTGGACTCGGAACAAG |
| mCyp7b1-Reverse | AATCGGGGTGCTGAATACCTAA |
| mGapdh-Forward | AGGTCGGTGTGAACGGATTTG |
| mGapdh-Reverse | TGTAGACCATGTAGTTGAGGTCA |
| mAr-Forward | AACCCATTGGACTACGGCAG |
| mAr-Reverse | GGAGACGACAAGATGGGCAA |
| mFkbp5-Forward | TGAGGGCACCAGTAACAATGG |
| mFkbp5-Reverse | CAACATCCCTTTGTAGTGGACAT |
| mTrpm8-Forward | CCAAGGAGTTTCCAACAGACG |
| mTrpm8-Reverse | CGTGGCTTCAAAGCAAAGTTT |
| mNkx3.1-Forward | CCGGAGGACCCACCAAGTAT |
| mNkx3.1-Reverse | CCTGGATTATGTTCACAGTCCAA |
| mSlc45a3-Forward | CGGCATTACCTATGTGCCAC |
| mSlc45a3-Reverse | GCCCAGATAAAGGGTCTCCG |
| mEzh2-Forward | AGTGACTTGGATTTTCCAGCAC |
| mEzh2-Reverse | AATTCTGTTGTAAGGGCGACC |
| mEno2-Forward | AGGTGGATCTCTATACTGCCAAA |
| mEno2-Reverse | GTCCCCATCCCTTAGTTCCAG |
| mChga-Forward | ATCCTCTCTATCCTGCGACAC |
| mChga-Reverse | GGGCTCTGGTTCTCAAACACT |
| mSyp-Forward | AGACATGGACGTGGTGAATCA |
| mSyp-Reverse | ACTCTCCGTCTTGTTGGCAC |
| mAscl1-Forward | GCAACCGGGTCAAGTTGGT |
| mAscl1-Reverse | CAAGTCGTTGGAGTAGTTGGG |
| mCps1-Forward | CAGTCAGCCTACAGCCTCAAC |
| mCps1-Reverse | CTCTTTGCGGTCACTCTCCA |
| mIgfbp5-Forward | CAGATTCCGAGACGCCTACC |
| mIgfbp5-Reverse | AGAGGACAGAGCTACGGTGT |
| mSidt2-Forward | CCTTTGCTGTTTGTGGTCCG |
| mSidt2-Reverse | GTGACCACTTTCTGGGCAGG |
| mTmem176b-Forward | CTGTGCCACCTTGGGTGTAT |
| mTmem176b-Reverse | GGAGACATGATACCTGGCCG |
| mCxcl17-Forward | GGCACTGCCTCACAGTGTAT |
| mCxcl17-Reverse | CGAGGTGTCCTGCATTCTTG |
| mXbp1-Forward | AGCAGCAAGTGGTGGATTTG |
| mXbp1-Reverse | GAGTTTTCTCCCGTAAAAGCTGA |
| mRetreg3-Forward | CCTATGTGCGACTGAAGCCA |
| mRetreg3-Reverse | GCCATCTAGGTCTGAGGACAG |
| mXbp1s-Forward | CTGAGTCCGCAGCAGGTG |
| mXbp1s-Reverse | GCCCAGTGTTATGTGGCTCT |
| Primers for shRNA | Sense and anti-sense sequence (5’ to 3’) |
| mCh25h-shRNA1-sense | GCGTGGTGTATTTGAATCATT |
| mCh25h-shRNA1-anti-sense | AATGATTCAAATACACCACGC |
| mCh25h-shRNA2-sense | CCACTCACCATCTTTACCTTT |
| mCh25h-shRNA2-anti-sense | AAAGGTAAAGATGGTGAGTGG |
| mCps1-shRNA1-sense | TGTCATCCGCCTGCTAGTTAA |
| mCps1-shRNA1-anti-sense | TTAACTAGCAGGCGGATGACA |
| mCps1-shRNA2-sense | AGTCTACTTTCTCCCTATTAC |
| mCps1-shRNA2-anti-sense | GTAATAGGGAGAAAGTAGACT |
| mIgfbp5-shRNA1-sense | GAGATGAGACAGGAATCCGAA |
| mIgfbp5-shRNA1-anti-sense | TTCGGATTCCTGTCTCATCTC |
| mIgfbp5-shRNA2-sense | CGACGAGAAAGCTCTGTCCAT |
| mIgfbp5-shRNA2-anti-sense | ATGGACAGAGCTTTCTCGTCG |
| mSidt2-shRNA1-sense | GCACGAAAGGACAAACGTGTT |
| mSidt2-shRNA1-anti-sense | AACACGTTTGTCCTTTCGTGC |
| mSidt2-shRNA2-sense | GCAGCGAGAGATCAATCATAA |
| mSidt2-shRNA2-anti-sense | TTATGATTGATCTCTCGCTGC |
| mTmem176b-shRNA1-sense | CGTGTCCCTGTCCATAGTAAT |
| mTmem176b-shRNA1-anti-sense | ATTACTATGGACAGGGACACG |
| mTmem176b-shRNA2-sense | AGTGCGATACAGTGATGATT |
| mTmem176b-shRNA2-anti-sense | AATCATCACTGTATCGCACTG |
| mCxcl17-shRNA1-sense | GCCAGCAATTTCTCAAACGAT |
| mCxcl17-shRNA1-anti-sense | ATCGTTTGAGAAATTGCTGGC |
| mCxcl17-shRNA2-sense | CTTTGCGCTGCCCTTATAGTA |
| mCxcl17-shRNA2-anti-sense | TACTATAAGGGCAGCGCAAAG |
| mXbp1-shRNA1-sense | CCATTAATGAACTCATTGGTT |
| mXbp1-shRNA1-anti-sense | AACCAATGAGTTCATTAATGG |
| mXbp1-shRNA2-sense | CCAGGAGTTAAGAACACGCTT |
| mXbp1-shRNA2-anti-sense | AAGCGTGTTCTTAACTCCTTGG |
| mRetreg3-shRNA1-sense | CATTCAGCTTGATGATCATTG |
| mRetreg3-shRNA1-anti-sense | CAATGATCATCAAGCTGAATG |
| mRetreg3-shRNA2-sense | TGTGAAAGTTAGGCATAATAA |
| mRetreg3-shRNA2-anti-sense | TTATTATGCCTAACTTTCACA |
| mGata2-shRNA1-sense | CCTGCAACACACCACCCGATA |
| mGata2-shRNA1-anti-sense | TATCGGGTGGTGTGTTGCAGG |
| mGata2-shRNA2-sense | CCCTGTAAATACAACCTTCTT |
| mGata2-shRNA2-anti-sense | AAGAAGGTTGTATTTACAGGG |
| mRest-shRNA1-sense | GTGTAATCTACAATACCATTT |
| mRest-shRNA1-anti-sense | AAATGGTATTGTAGATTACAC |
| mRest-shRNA2-sense | CGCGGCTTCTAAGAAGTGTAA |
| mRest-shRNA2-anti-sense | TTACACTTCTTAGAAGCCGCG |
| mFoxa1-shRNA1-sense | GCTGTCCTTCAACGATTGTTT |
| mFoxa1-shRNA1-anti-sense | AAACAATCGTTGAAGGACAGC |
| mFoxa1-shRNA2-sense | GTATTCCAGACCCGTGCTAAA |
| mFoxa1-shRNA2-anti-sense | TTTAGCACGGGTCTGGAATAC |
| mStat3-shRNA1-sense | CCTGAGTTGAATTATCAGCTT |
| mStat3-shRNA1-anti-sense | AAGCTGATAATTCAACTCAGG |
| mStat3-shRNA2-sense | CGACTTTGCTTTCAACTACAA |
| mStat3-shRNA2-anti-sense | TTGTAGTTGAAATCAAAGTCG |
| mPtk2-shRNA1-sense | CCTGGCATCTTTGATATTATA |
| mPtk2-shRNA1-anti-sense | TATAATATCAAAGATGCCAGG |
| mPtk2-shRNA2-sense | GCCTTAACAATGCGTCAGTTT |
| mPtk2-shRNA2-anti-sense | AAACTGACGCATTGTTAAGGC |
| Primers for Xbp1 promoter probe | Sequence (5’ to 3’) |
| mXbp1-biotin F | GAGATGGCTCAGTGGTTAAGAACACTGA |
| mXbp1-R | AGTCCAAGGATCTTCGGCAGACG |
| mXbp1-cold F | GAGATGGCTCAGTGGTTAAGAACACTGA |
| Primers for ChIP-qPCR | Sequence (5’ to 3’) |
| mXbp1-bs1-F | GGAGATGTTGTACCTAGTCCTGCT |
| mXbp1-bs1-R | CTCCATGGTCTGCTTGTCTCTG |
| mXbp1-bs2-F | GGCCTAGCTAGACCTCCCTATGT |
| mXbp1-bs2-R | TACCCTAGCCCGACCCCATT |
| hXbp1-bs1-F | CCCATCCTGTTCCCTCCCTAC |
| hXbp1-bs1-R | GTGGCCAGGTGTTTGTGAAAGAGG |
| hXbp1-bs2-F | CTGTCCAGTCTTTTCGAACCCAAG |
| hXbp1-bs2-R | CGTTTCAGGACCGTGGCTAT |
| Oligonucleotides | Sequence (5’ to 3’) |
| siCh25h-1-F | UGGGACACCAUAAGGACAATT |
| siCh25h-1-R | UUGUCCUUAUGGUGUCCCATT |
| siCh25h-2-F | CGACCCAAUACAUGAGCUUTT |
| siCh25h-2-R | AAGCUCAUGUAUUGGGUCGTT |
