# Supplementary figures and images for: Cholesterol‐Mediated Metabolic‐mechanotransductive Crosstalk Orchestrates Castration Resistance in Prostate Cancer
Source: Adv Sci (Weinh). 2026 Jun 4:e75977. Online ahead of print. doi: 10.1002/advs.75977 (PMC13336987; doi:10.1002/advs.75977)

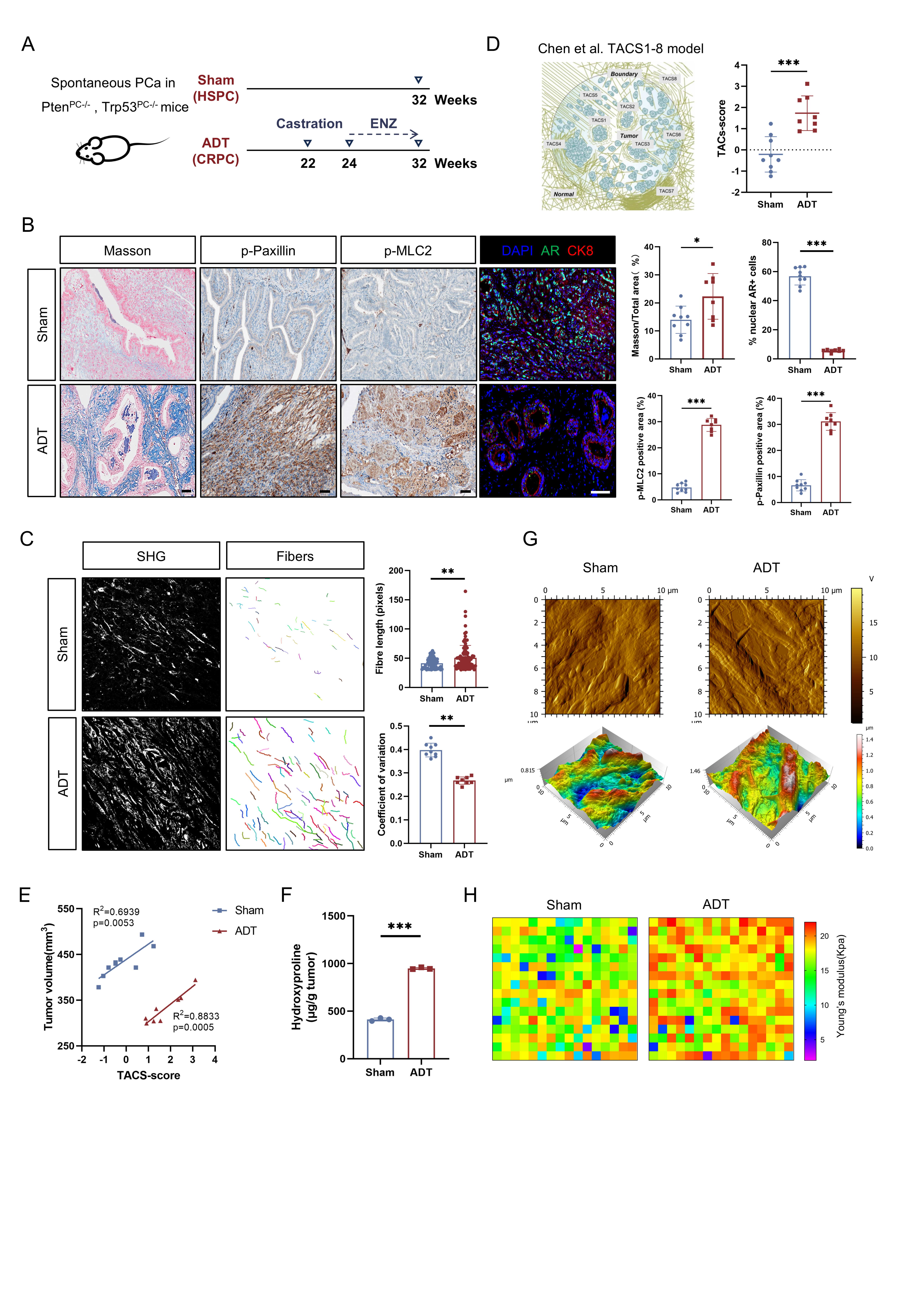

Supplement: Supplementary file 2 — Supporting File: advs75977‐sup‐0002‐FigureS1‐S7.zip. [file ADVS-9999-e75977-s001.zip › Figure S1.tif]

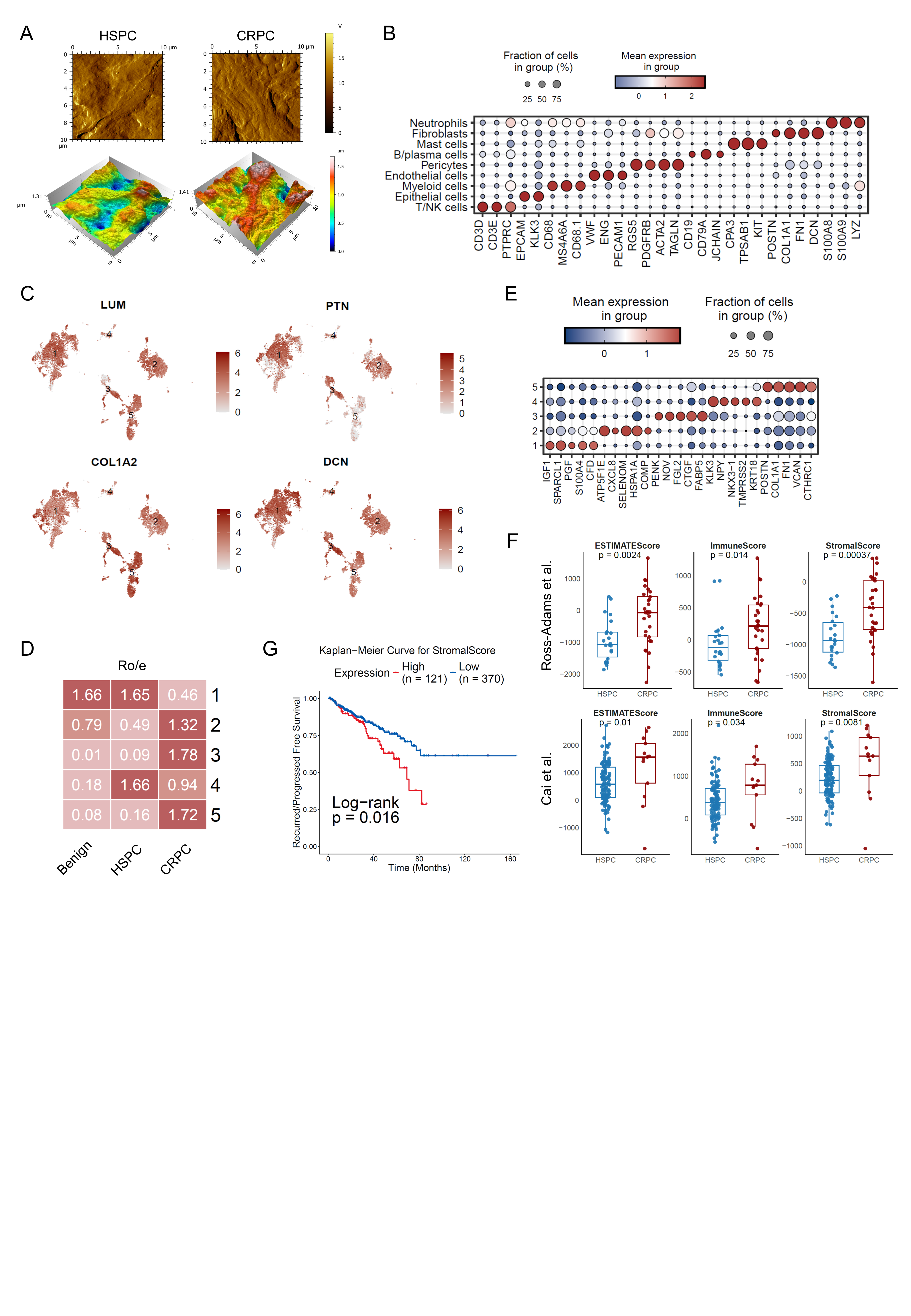

Supplement: Supplementary file 2 — Supporting File: advs75977‐sup‐0002‐FigureS1‐S7.zip. [file ADVS-9999-e75977-s001.zip › Figure S2.tif]

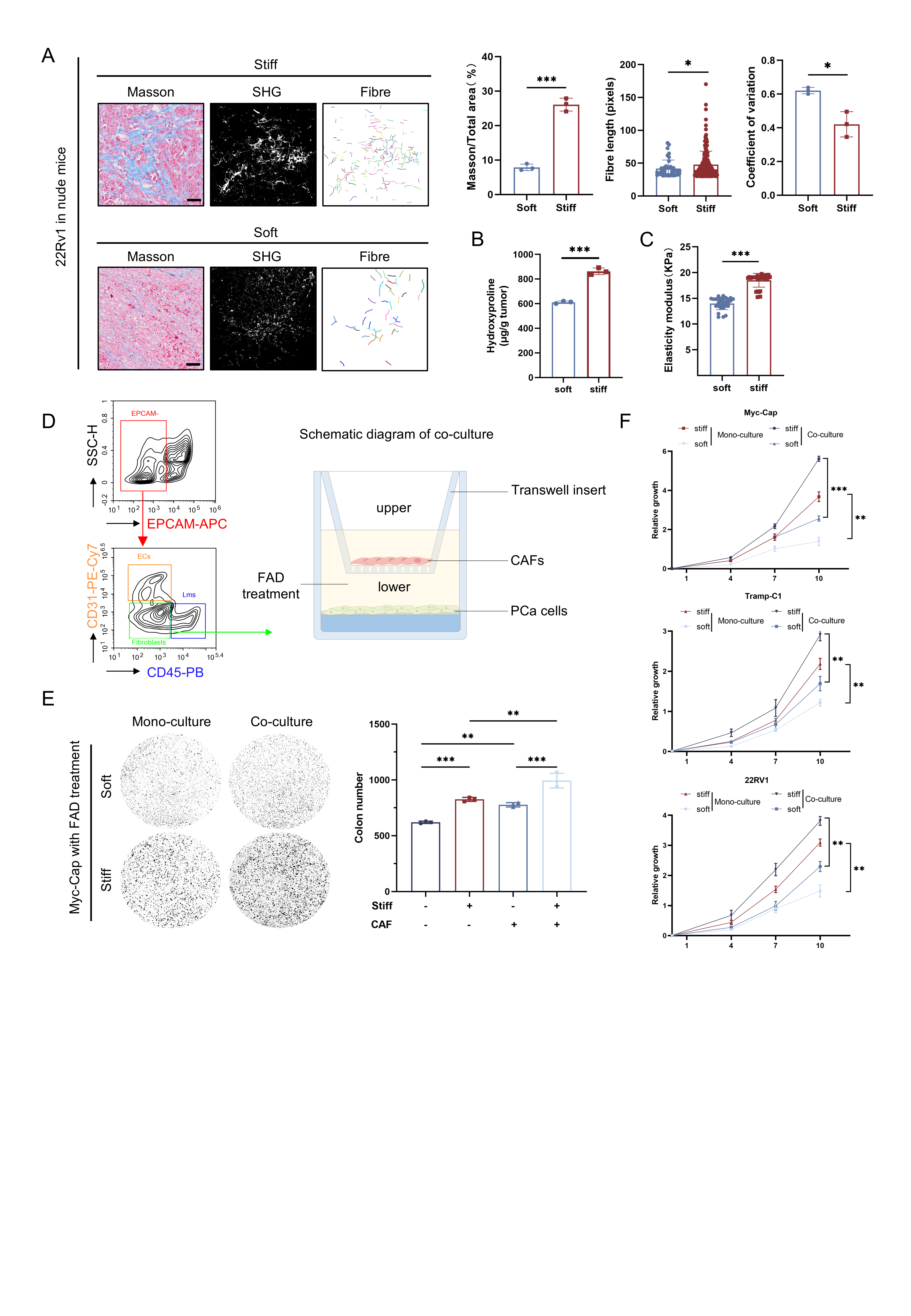

Supplement: Supplementary file 2 — Supporting File: advs75977‐sup‐0002‐FigureS1‐S7.zip. [file ADVS-9999-e75977-s001.zip › Figure S3.tif]

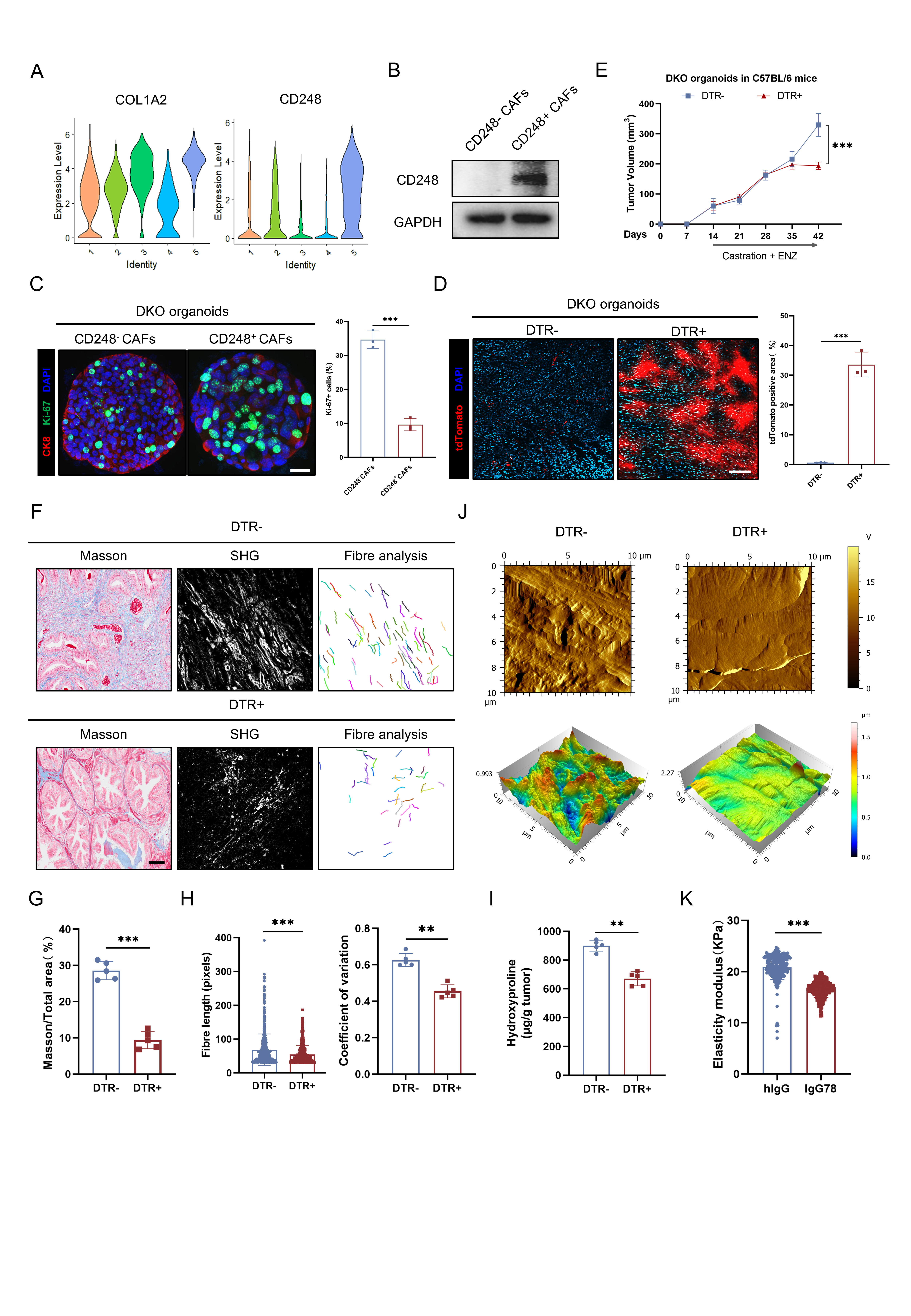

Supplement: Supplementary file 2 — Supporting File: advs75977‐sup‐0002‐FigureS1‐S7.zip. [file ADVS-9999-e75977-s001.zip › Figure S4.tif]

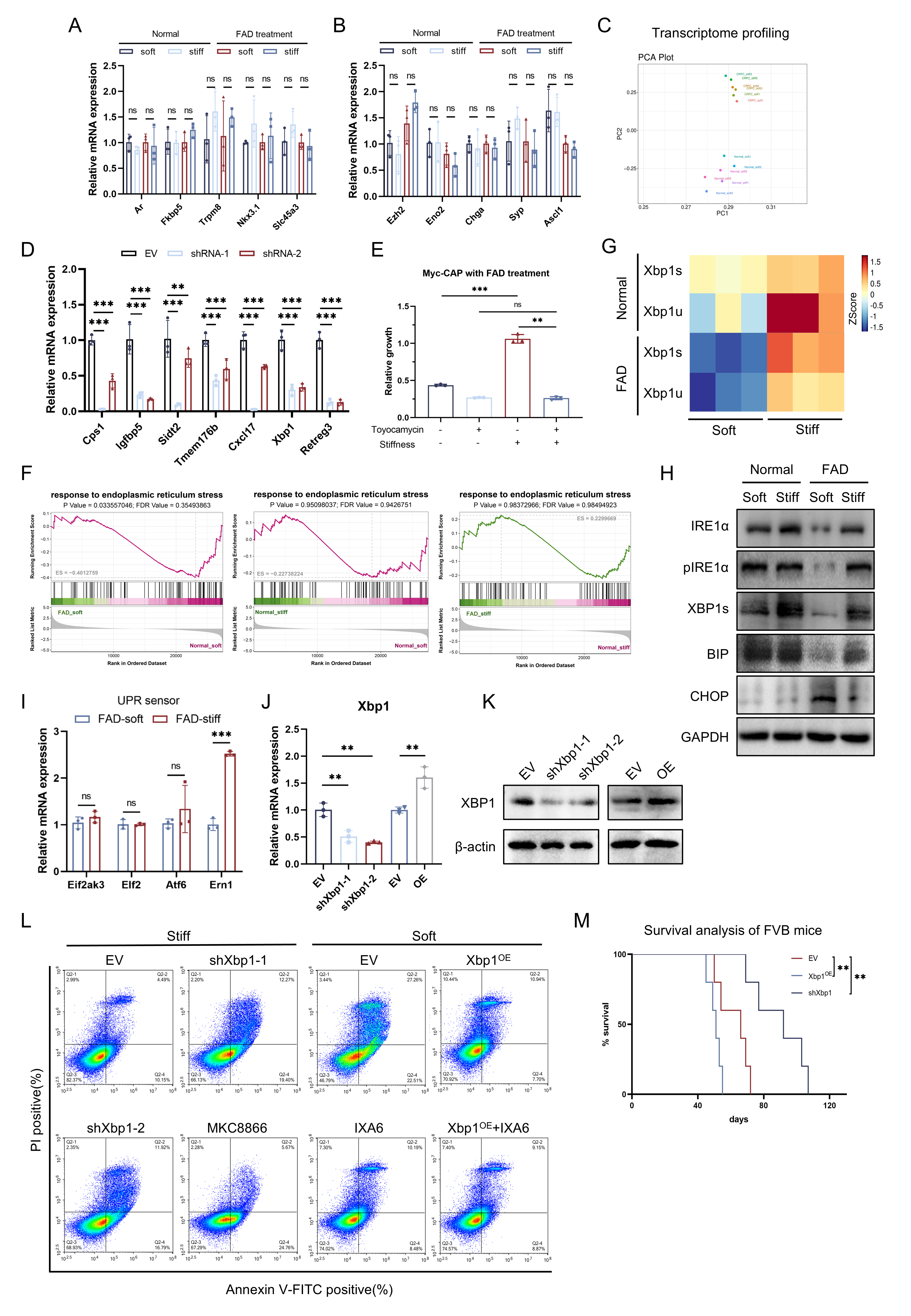

Supplement: Supplementary file 2 — Supporting File: advs75977‐sup‐0002‐FigureS1‐S7.zip. [file ADVS-9999-e75977-s001.zip › Figure S5.tif]

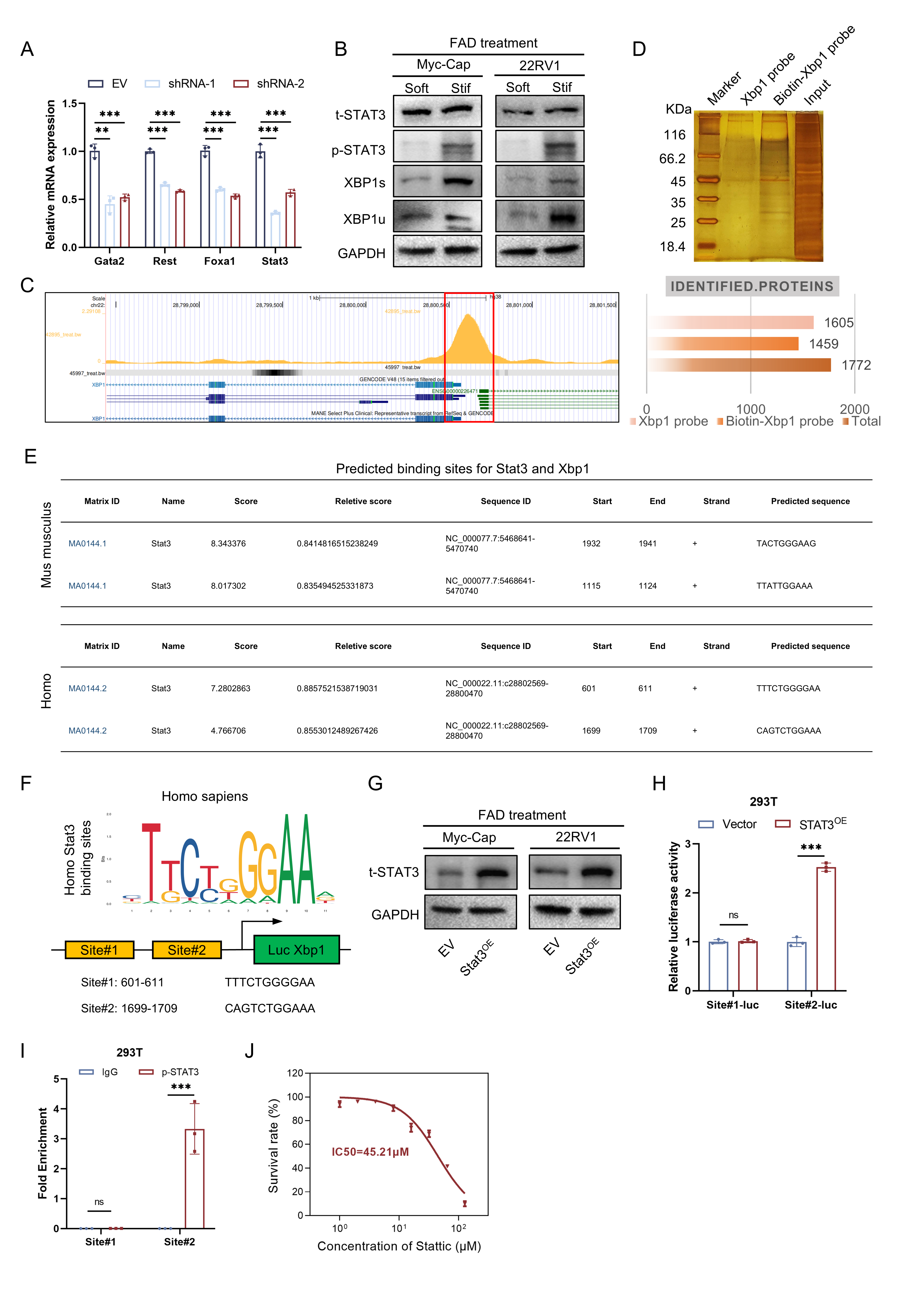

Supplement: Supplementary file 2 — Supporting File: advs75977‐sup‐0002‐FigureS1‐S7.zip. [file ADVS-9999-e75977-s001.zip › Figure S6.tif]

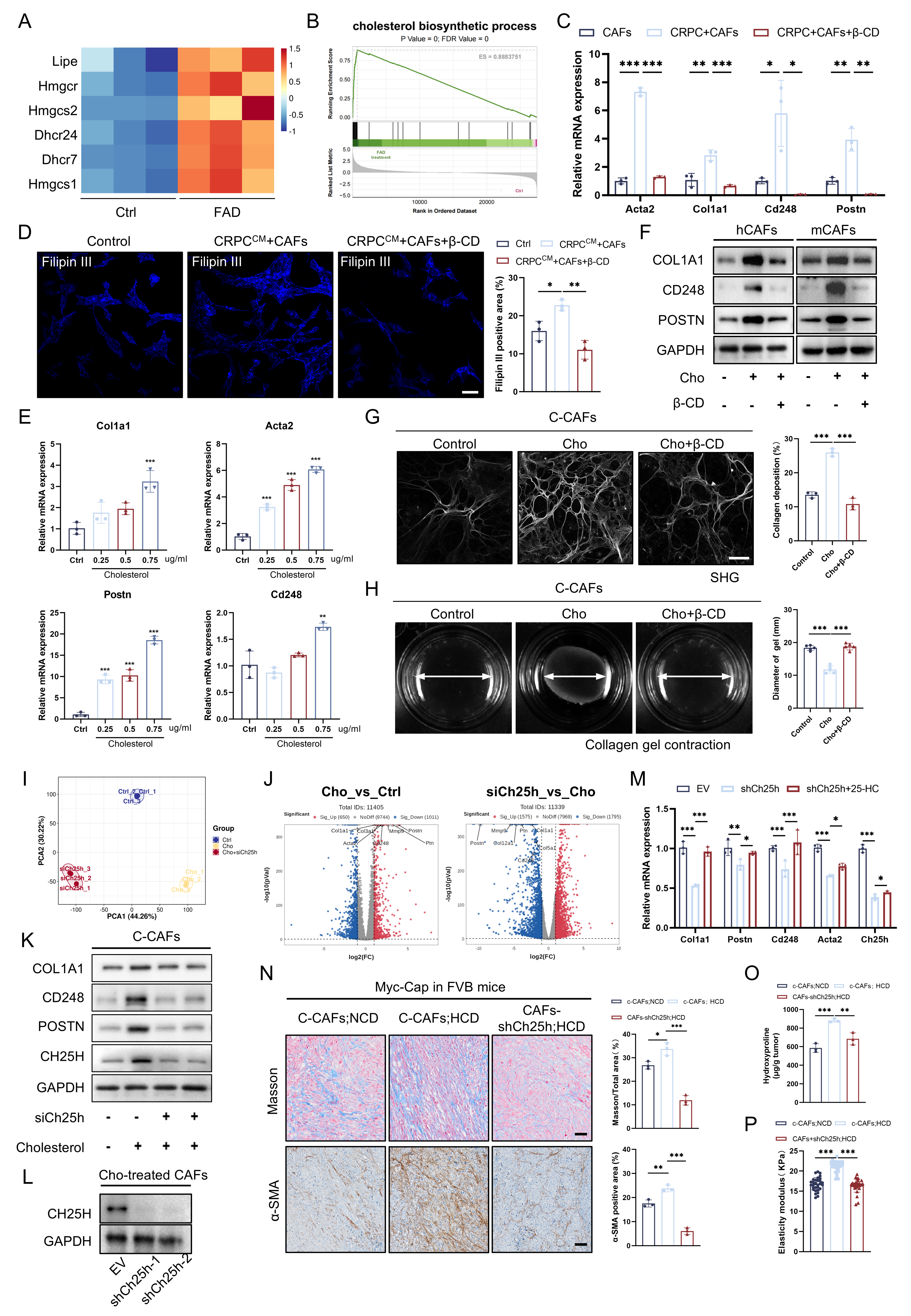

Supplement: Supplementary file 2 — Supporting File: advs75977‐sup‐0002‐FigureS1‐S7.zip. [file ADVS-9999-e75977-s001.zip › Figure S7.tif]
